# Boroles from alumoles: accessing boroles with alkyl-substituted backbones *via* transtrielation†

**DOI:** 10.1039/d3sc02668j

**Published:** 2023-07-19

**Authors:** Josina L. Bohlen, Lukas Endres, Regina Drescher, Krzysztof Radacki, Maximilian Dietz, Ivo Krummenacher, Holger Braunschweig

**Affiliations:** a Institute for Inorganic Chemistry, Julius-Maximilians-Universität Würzburg Am Hubland 97074 Würzburg Germany h.braunschweig@uni-wuerzburg.de; b Institute for Sustainable Chemistry & Catalysis with Boron, Julius-Maximilians-Universität Würzburg Am Hubland 97074 Würzburg Germany

## Abstract

The alumole Cp^3t^AlC_4_Et_4_ (Cp^3t^ = 1,2,4-tris(*tert*-butyl)cyclopentadienyl) is reported to be capable of transferring its butadiene moiety to aryl(dihalo)boranes to generate boroles through aluminum–boron exchange. The products feature a rare alkyl-substituted backbone, which, as shown in other examples, often leads to dimerization due to insufficient steric protection of the antiaromatic borole ring. Sterically crowded aryl groups bound to the boron atom are shown to prevent dimerization, allowing access to the first monomeric derivatives of this type. Results from UV-vis spectroscopy, electrochemistry, and DFT calculations reveal that the alkyl substituents cause remarkable modifications in the optical and electronic properties of the boroles compared to their perarylated counterparts.

## Abstract 2

The synthesis of monomeric 2,3,4,5-tetraalkyl-substituted boroles *via* metallacycle transfer from an alumole is reported.

## Graphical abstract

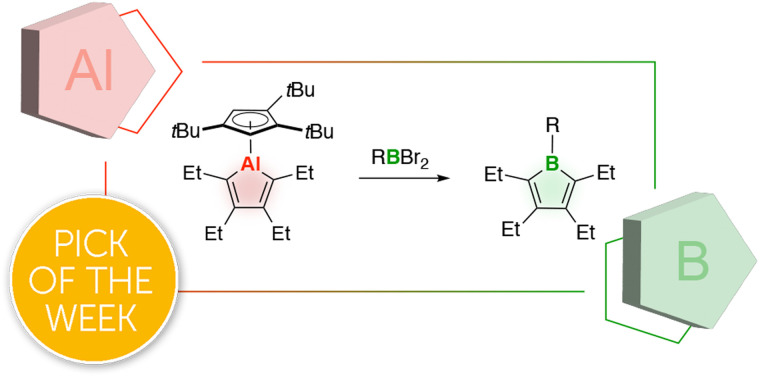

## Introduction

The transfer of organic ligands from one metal to another, known as transmetalation, is an important organometallic reaction with great utility in synthetic chemistry.^1^ Not only is it a crucial step in Nobel Prize-winning palladium-catalyzed cross-coupling reactions,^2^ it also enables the preparation of a variety of organometallic compounds of both main group and transition metals.^1^ For instance, this strategy has greatly increased the synthetic accessibility of unsaturated five-membered organometallic heterocycles, so called metalloles,^3^ which are increasingly finding applications as light-emitting materials due to their unique optoelectronic properties.^4^ In the most common reaction process, the metalloles are generated by reacting zirconacyclopentadienes with metal or p-block element halides.^5^ The reaction, named after its developers Fagan and Nugent, is characterized by high efficiency and great generality, especially in the preparation of five-membered heterocycles of the heavier p-block elements.^5^ Lighter analogues such as boroles are also accessible, but the reaction often fails, and so far only very few examples have been described.^6^

**Scheme 1:**
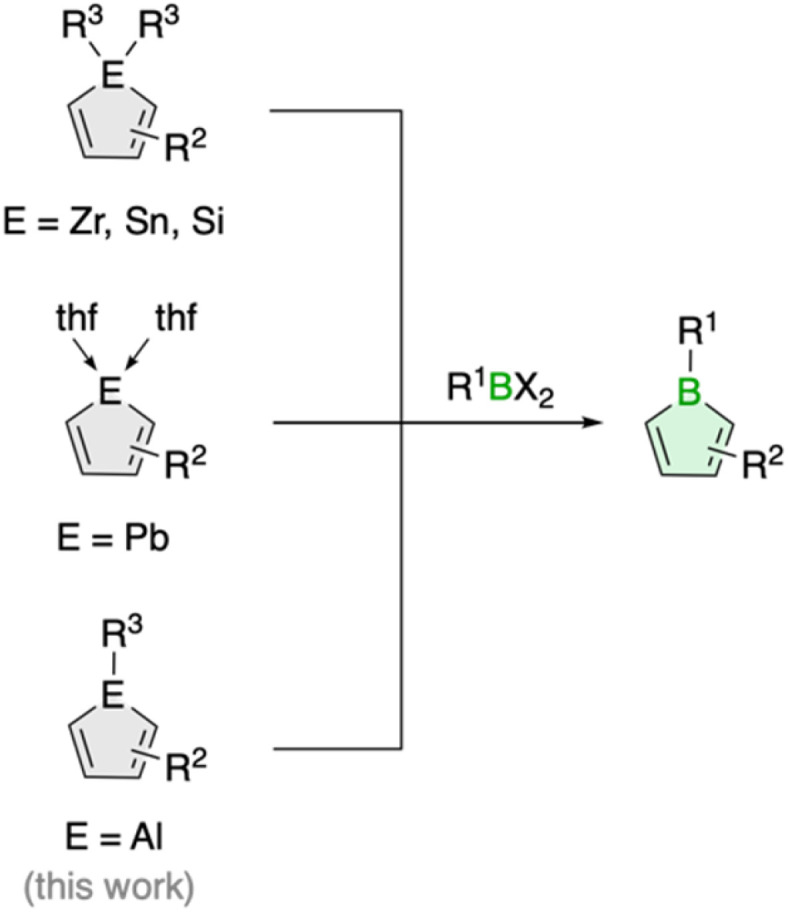
Generic scheme for borole synthesis *via* metallacycle transfer.

Boroles, which have a distinct antiaromatic character, are generally more readily available from other metalloles, such as stannoles.^7^ Besides the cyclization of 1,4-dilithio-1,3-dienes with (organo)boron halides, transmetalation *via* tin–boron exchange is probably the most common method for the synthesis of monocyclic boroles, especially for derivatives with aryl groups in the carbon backbone.^7^ Further metallacycle transfers to boron have been reported for silicon analogues (Scheme 1).^8^ These routes are particularly valuable for the preparation of dibenzannulated boroles, also known as 9-borafluorenes.^9^ Moreover, a unique transmetalation of a plumbole with boron trifluoride etherate was described,^10^ yielding a borole derivative with a B–F bond that was not accessible by other methods.^11^ It further featured silyl groups at the 2,5 positions, substituents known to electronically interfere with the ability of stannoles to transfer their diene moiety.^12^

Such alternative transmetalation reactions may thus prove advantageous for the introduction of new functionalities around the borole ring that can lead to new properties. Herein, we show that alumoles, the heavier group 13 homologues of boroles,^13^ can serve as effective precursors for the preparation of boroles with alkyl substituents in the backbone. Alkyl-substituted borole derivatives are rare and have only been observed in equilibrium with their dimeric Diels–Alder cycloadducts.^6^ By incorporating ethyl substituents at the borole backbone and a bulky organic substituent at boron, we were able to obtain the first examples of monomeric 2,3,4,5-tetraalkylboroles. Moreover, we investigated the influence of the alkyl substituents on the chemical and physical properties of the boroles with a combination of spectroscopic, electrochemical and computational methods.

## Results and discussion

### Borole synthesis from alumoles

We have recently introduced a new alumole derivative (1) with a bulky 1,2,4-tris(*tert*-butyl)cyclopentadienyl (Cp^3t^) ligand at the aluminum atom, which exists as a monomer both in solution and in the solid state.^14^ Its properties are largely tied to the fluxional behavior of the Cp ligand, which ensures accessibility to the electrophilic Al center.^15^ We reasoned that the lower electronegativity of aluminum compared to boron and the lability of the aluminum–carbon bonds might lead to facile transfer of the diene unit to the boron atom.^16^

**Fig. 1:**
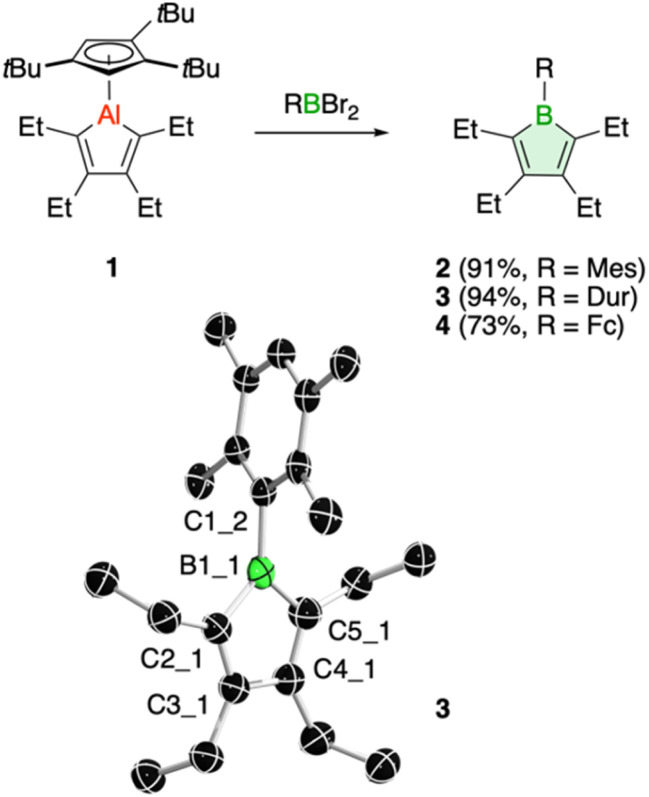
Borole synthesis from alumoles and molecular structure of 3 (ellipsoids at 50% probability). Selected bond lengths (Å) and angles (°): B1_1–C1_2 1.603(6), B1_1–C2_1 1.558(7), B1_1–C5_1 1.579(8), C2_1–C3_1 1.349(6), C3_1–C4_1 1.522(7), C4_1–C5_1 1.342(7); C5_1–B1_1–C2_1 104.5(4).

**Fig. 2:**
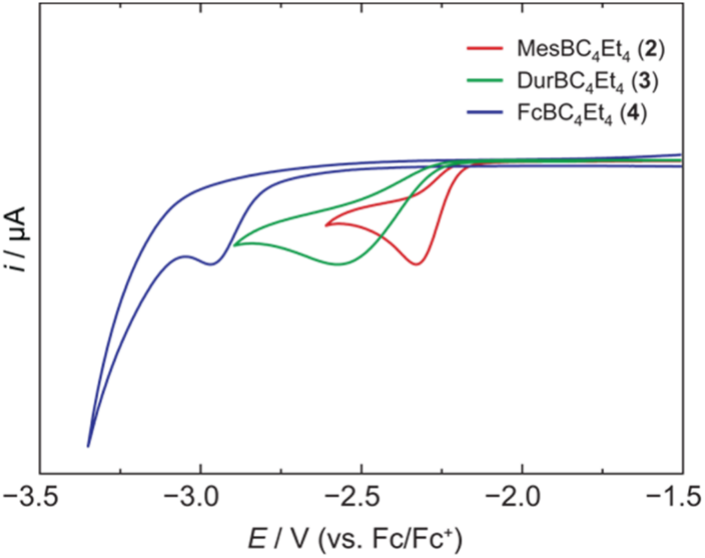
Cyclic voltammograms of 2, 3, and 4 in dichloromethane solution showing the first reduction events (supporting electrolyte: [NBu_4_][PF_6_] (0.1 M), scan rate: 250 mV s^−1^).

Treatment of 1 with the sterically hindered aryl(dibromo)boranes MesBBr_2_ (Mes = 2,4,6-trimethylphenyl)^17^ and DurBBr_2_ (Dur = 2,3,5,6-tetramethylphenyl)^18^ led to no immediate reaction in benzene solutions, but a slow conversion to a new product was observed at higher temperatures. The new broad ^11^B NMR resonances at *δ*(^11^B) = 76.5 (2) and 77.6 ppm (3), respectively, are consistent with formation of the expected borole rings (Fig. 1).^7^ Despite full conversion of alumole 1, isolation of the boroles proved challenging because of the difficulty in separating the hydrocarbon-soluble aluminum species Cp^3t^AlBr_2_, which is formed as a side product. After numerous attempts to induce precipitation of the aluminum compound by adduction formation, we found that the addition of the cyclic(alkyl)(amino)carbene CAAC^Me^ (1-(2,6-diisopropylphenyl)-3,3,5,5-tetramethylpyrrolidin-2-ylidene)^19^ results in an insoluble adduct that can be easily removed by filtration. By this process, the corresponding air-sensitive boroles were obtained in analytically pure form in yields exceeding 90% (Fig. 1).‡‡We found that certain borole samples contained small paramagnetic impurities that likely originate from soluble CAAC-stabilized aluminum radicals. Despite the oily nature of the products, we were able to obtain crystals suitable for X-ray diffraction analysis in the case of 3 (Fig. 1). Although problems with refinement (*i.e.* disorder across the crystallographic mirror plane) preclude a detailed analysis of the complete structure, the solution is sufficient to discuss the bond parameters of the central borole ring (see ESI† for details). The alternating single and double carbon–carbon bond lengths of 1.342(7), 1.522(7), and 1.349(6) Å show no or only small differences to those of the perarylated borole MesBC_4_Ph_4_; although the C–C single bond is statistically shorter (1.522(7) *vs.* 1.560(2) Å).^20^ The boron-carbon bonds as well as the 73.6° twist of the duryl group relative to the borole ring are likewise comparable. The effect of the ethyl substituents on the conjugation in the ring is thus not readily apparent, which may also be due – at least in part – to the greater uncertainty in the bond lengths of 3. Boroles 2 and 3 appear red, corresponding to light absorption in the green region of the spectrum (*λ*_max_ = 505 nm and *λ*_max_ = 500 nm, respectively). The UV-vis absorptions are thus significantly blue-shifted compared to the perarylated boroles PhBC_4_Ph_4_ (*λ*_max_ = 560 nm)^21^ and MesBC_4_Ph_4_ (*λ*_max_ = 578 nm),^20^ which are blue and green solids, respectively. These lowest energy absorptions are associated with HOMO–LUMO transitions, demonstrating that the alkyl-substituted backbone in 2 and 3 is what causes the larger optical band gap.^22^ Cyclic voltammetry, which provides a way to estimate the LUMO energy through the measurement of the reduction potential of a molecule, allowed a better understanding of the relative energies of the frontier orbitals. Boroles 2 and 3 were found to undergo irreversible reduction events at −2.33 and −2.57 V *vs.* Fc/Fc^+^, respectively, which are cathodically shifted by more than 0.6 V relative to those of PhBC_4_Ph_4_ (*E*_1/2_ = −1.61 V *vs.* Fc/Fc^+^)^23^ and MesBC_4_Ph_4_ (*E*_1/2_ = −1.69 V *vs.* Fc/Fc^+^, Fig. 2).^20^ The more negative reduction potentials of the alkyl-substituted boroles indicate that their LUMOs are significantly higher in energy than those of the aryl-substituted boroles, contributing to the larger HOMO–LUMO gaps (see DFT results below). To the best of our knowledge, boroles 2 and 3 represent the first monocyclic borole derivatives with alkyl substituents on the diene backbone. Previous derivatives were shown to undergo spontaneous Diels–Alder dimerization due to insufficient steric shielding by the ring substituents (*c.f.* PhBC_4_Me_4_ or PhBC_4_Et_4_).^6^

A range of other dihaloboranes was evaluated as reagents to assess the scope of the transmetalation reaction. We found that boroles 2 and 3 could also be obtained by using dichloroboranes instead of the dibromoboranes MesBBr_2_ and DurBBr_2_, respectively. In a similar manner, (dibromoboryl)ferrocene (FcBBr_2_)^24^ reacts cleanly with alumole 1 to afford the borole product 4 (73% yield; Fig. 1). In this case, the transformation proceeds rapidly at room temperature without the need for heating. Because of its propensity to form oils, borole 4 was not readily susceptible to crystallization and was instead isolated as a dark red oil. The ^11^B NMR resonance at *δ*(^11^B) = 54.7 is consistent with the presence of an intramolecular iron–boron interaction in borole 4, as observed in many other borylferrocenes including FcBC_4_Ph_4_ (*δ*(^11^B) = 47.4 ppm).^21,25^ As expected from this stabilizing donor–acceptor interaction, the boron atom in 4 becomes less electrophilic and is therefore reduced at more negative potential (*E*_pc_ = −2.97 V) than its *B*-aryl substituted analogues 2 and 3 (Fig. 2). Borole 4 has been further characterized by high-resolution mass spectrometry (HRMS), multinuclear NMR and UV-vis spectroscopy. The UV-vis spectrum of 4 in dichloromethane solution shows two absorption bands at *λ*_max_ = 472 and 372 nm, which are again blue-shifted compared to those of the phenyl-substituted counterpart (FcBC_4_Ph_4_: *λ*_max_ = 490 and 390 nm).^7*d*,21^ Similar to boroles 2 and 3, borole 4 does not form an adduct with thf.

**Fig. 3:**
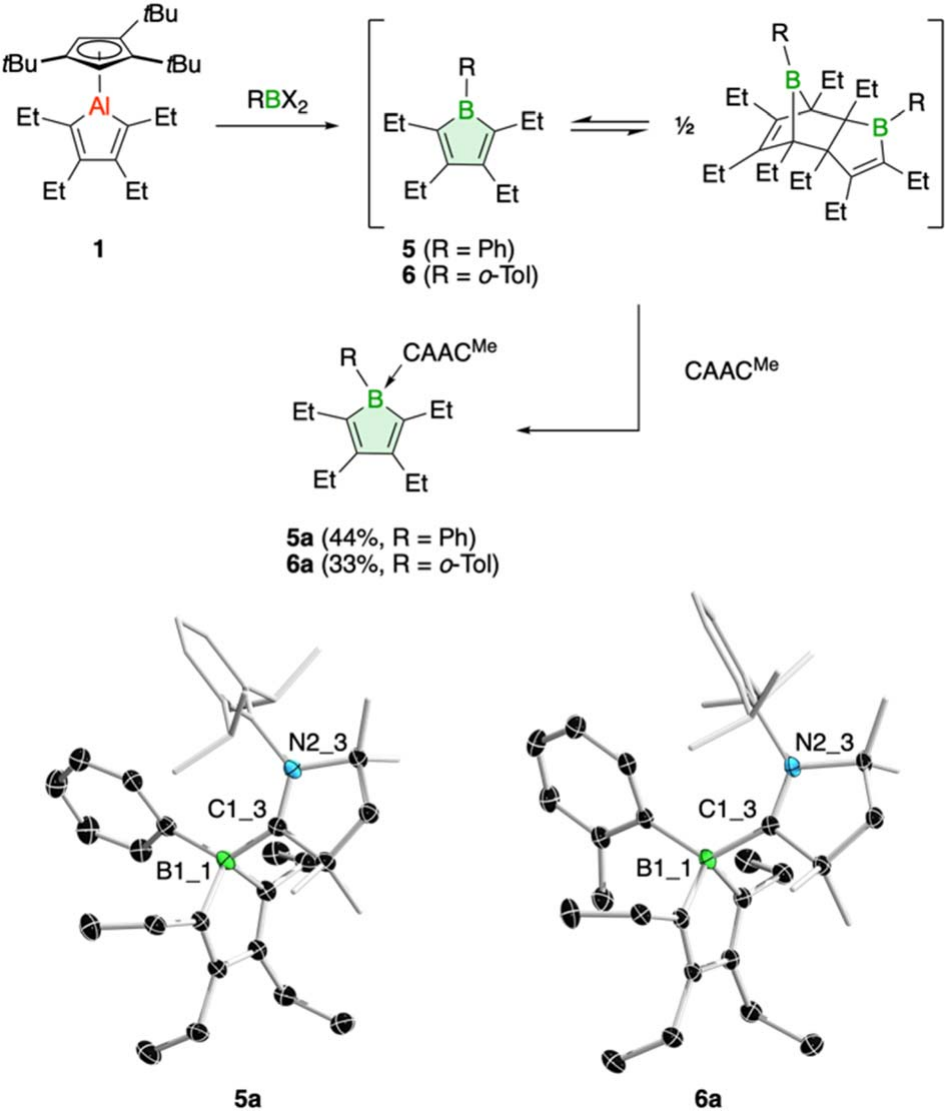
Synthesis of borole adducts 5a and 6a through trapping reactions of the borole monomers 5 and 6, respectively (top). Molecular structures of 5a and 6a with displacement ellipsoids shown at 50% probability (bottom; H atoms omitted for clarity).

In addition, we have tested the aluminum–boron exchange reaction to access the phenyl-substituted derivative PhBC_4_Et_4_ (5), previously reported by Martin and coworkers.^6*c*^ The compound was isolated as its Diels–Alder dimer, but in solution was shown to be in equilibrium with its monomeric variant. Using our protocol, addition of dibromo(phenyl)borane to 1 resulted in complete conversion of the alumole within a few minutes at room temperature (Fig. 3). After work up using CAAC^Me^ to remove the aluminum by-product, we obtained borole 5 as carbene adduct 5a in 44% yield. The base-free borole 5 could not be isolated cleanly. The orange adduct was amenable to crystallization. Its molecular structure, as determined by single-crystal X-ray diffraction, shows no unusual features compared to other carbene-borole adducts, with unobtrusive B–C(carbene) (1.654(2) Å), endocyclic B–C (av. 1.651(3) Å), single (1.485(2) Å) and double C–C (av. 1.353(3) Å) bond lengths.^26^ In addition, the ^11^B NMR signal at *δ* = −3.9 ppm is in a typical range seen for other borole adducts.^26^ The isolation of borole adduct 5a reaffirms that the Diels–Alder dimer of 5 exists in solution in equilibrium with its monomer.^6*c*^

Finally, one last variation in the steric demand of the aryl group in the dihaloborane reagent was explored. Dichloro(*ortho*-tolyl)borane^27^ was reacted with alumole 1 in benzene, resulting in immediate formation of a purple solution. Unfortunately, standard workup with one equivalent of the carbene CAAC^Me^ yielded only an impure product, contaminated with the aluminum species Cp^3t^AlCl_2_ and the carbene adduct of the expected borole 6. Numerous attempts to isolate 6 in pure form proved unsuccessful. In the process, we found that utilizing bromide instead of chloride in the borane reagent did not bring about a successful aluminum–boron exchange reaction. We thus added two equivalents of the carbene to selectively obtain borole adduct 6a (Fig. 3). Despite the *ortho*-methyl substituent on the aryl group, adduct formation proceeds smoothly and 6a can be obtained in 33% yield as an orange-red solid. The transformation is reflected in a characteristic low-frequency ^11^B NMR signal at *δ*(^11^B) = −2.7 ppm, consistent with a tetrahedral boron atom.^7,26^ The Lewis acid-base structure was revealed by X-ray diffraction analysis and consists of a boron–carbene bond of 1.680(2) Å.^26^ The borole intraring distances are comparable to those of 5a.

### Tin–boron exchange

To determine if the aluminum–boron exchange has an advantage over the tin–boron exchange, we also attempted to synthesize the new alkyl boroles *via* this more conventional approach. The comparison revealed some interesting differences between the two methods.

While the synthesis of boroles 2, 4, and 6 from the stannole Me_2_SnC_4_Et_4_ succeeded,^28^ it was unsuccessful for the duryl-(3) and phenyl-substituted derivatives (5), using both dichloro- and dibromoboranes. The successful transformations occurred with excellent yields of over 90% (detailed synthetic procedures can be found in the ESI†). In direct comparison, alumole 1 appears to be equally effective as the stannole in transferring the 1,2,3,4-tetraethyl-1,3-butadiene-1,4-diyl fragment for the formation of boroles. However, the use of the Al–B exchange method is accompanied by the formation of highly soluble aluminum dihalides as by-products, which is a drawback to consider, especially in light of the additional use of CAAC^Me^ and the synthesis of alumole 1. The presence of these aluminum species hindered the isolation of the *o*-tolyl derivative 6. However, in this case, the tin–boron exchange reaction proved effective, allowing us to isolate and characterize the free borole 6. We found that it exists in solution in both monomeric and dimeric forms that interconvert on the NMR timescale. At 70 °C, the equilibrium shifts almost completely to the side of the monomer (*δ*(^11^B) = 74.6 ppm). At lower temperatures, a characteristic ^11^B NMR signal for the dimer is detectable at −11.3 ppm, while the signal for the non-bridgehead boron atom is masked by the monomer signal.^6^ The shift of the equilibrium towards the monomeric form at higher temperatures is also visually evident as the color of the solution gradually intensifies to a deeper shade of purple. Furthermore, the monomeric borole form is readily detectable in the mass spectrum (*m*/*z* = 266.2195). No single crystals suitable for X-ray diffraction analysis could be obtained from either the monomeric or dimeric forms.

### DFT calculations

**Fig. 4:**
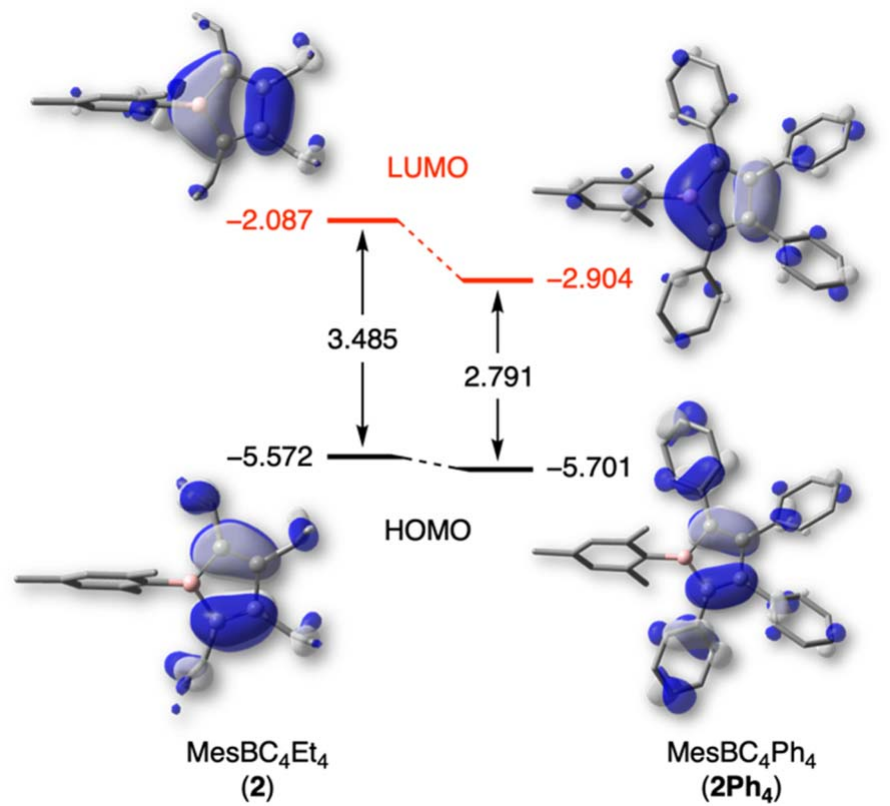
Frontier molecular orbitals and relative energies (in eV) for 2 and 2Ph_4_, calculated at the PBE0-D3(BJ)/6-311+G(d,p) level of theory.

To better understand the effects of the alkyl substituents on the nature and relative energies of the frontier orbitals, DFT calculations were undertaken. 1-Mesityl-2,3,4,5-tetraphenylborole (MesBC_4_Ph_4_, abbreviated as 2Ph_4_ in the following),^20^ which has the same boron substituent as borole 2 but instead a tetraphenyl-substituted backbone, was as an ideal candidate for comparison. The PBE0-D3(BJ) functional^29^ was chosen because it provides geometric parameters that agree well with those obtained from X-ray diffraction analysis of 2Ph_4_. While experimental data are lacking for 2, the geometrically optimized structure of 2 shows no significant differences in bond lengths or angles in the planar BC_4_ ring compared to 2Ph_4_ (see Table S1 in the ESI†). The alternating carbon–carbon single (1.531 Å) and double bonds (1.353 Å) are also in line with the structural study of the duryl derivative 3. The most striking differences between the two boroles were found in the energy of their frontier orbitals, which are displayed in Fig. 4. The ethyl groups on the borole ring in 2 considerably increase the LUMO energy compared to 2Ph_4_ and thus also increase the HOMO–LUMO gap. The presence of tetraphenyl substitution in the backbone of compound 2Ph_4_ leads to a more delocalized LUMO, extending into the π systems of the phenyl rings. This extension leads to a greater stabilization of its LUMO compared to that of 2.^12^ The difference in the relative orbital energies of the two boroles can also be attributed to the different inductive effects of the backbone substituents. The phenyl groups, for which resonance conjugation with the borole ring is impaired due to their propeller-like arrangement, exert a negative inductive effect, while the ethyl groups exert a positive inductive effect. As a result, the energies of the frontier orbitals of 2 are further destabilized compared to 2Ph_4_. The differences in the relative energies of the orbitals are consistent with the electrochemical and UV-vis absorption data of 2, indicating a higher LUMO level and larger band gap, respectively, compared to 2Ph_4_.

Furthermore, natural bond orbital (NBO) analysis^30^ shows that the inductive electron donation from the ethyl substituents only marginally reduces the positive charge of the boron atom in 2 (+1.017) compared to that of 2Ph_4_ (+1.034). Similarly, the negative charge on the carbon atoms in the BC_4_ ring is only slightly higher in 2 (see ESI† for details). As evident from nucleus-independent chemical shift (NICS) calculations,^31^ the more electron-rich alkyl backbone in 2 results in a somewhat more antiaromatic borole than 2Ph_4_; borole 2 has a NICS(0) value of 16.38 ppm, while that for 2Ph_4_ is 14.75 ppm.^9*b*,32^ The NICS values calculated at larger distances from the ring center, primarily measuring the contribution from the π-system, come to the same conclusion (see ESI† for NICS_zz_ scan profiles).

Taken together, the ethyl substituents significantly perturb the electronic structure of the borole relative to perarylated derivatives. These ethyl-substituted boroles become significantly more electron rich, resulting in a higher LUMO energy level and a larger HOMO–LUMO gap.

## Conclusions

In summary, we have developed a protocol for borole synthesis *via* aluminum–boron exchange. Starting from a 2,3,4,5-tetraethyl-substituted alumole, the procedure enabled the synthesis of several borole derivatives with alkyl substituents in the backbone, including the first monomeric examples. Computational and experimental data indicate that they are inferior acceptors and have a larger HOMO–LUMO gap than their perarylated borole derivatives. Our work thus illustrates how the diene substituents can be used to modify the optical and electronic characteristics of boroles.

## Data availability

The data that supports the findings of this study are available on reasonabe request from the corresponding author.

## Author contributions

J. L. B. synthesized and characterized the compounds and carried out spectroscopic studies. L. M. completed the DFT calculations. R. D. performed preliminary studies. K. R. and M. D. performed X-ray crystallographic analyses. I. K. performed electrochemical experiments and wrote the original draft of the manuscript, which was edited by all authors. H. B. conceived and supervised the research.

## Conflicts of interest

There are no conflicts of interest to declare.

## Supplementary Material

SC-014-D3SC02668J-s001

SC-014-D3SC02668J-s002

## References

[cit1] Partyka D. V. (2011). Chem. Rev..

[cit2] Johansson Seechurn C. C. C., Kitching M. O., Colacot T. J., Snieckus V. (2012). Angew. Chem., Int. Ed..

[cit3] Dubac J., Laporterie A., Manuel G. (1990). Chem. Rev..

[cit4] Cai Y., Qin A., Tang B. Z. (2017). J. Mater. Chem. C.

[cit5] Fagan P. J., Nugent W. A., Calabrese J. C. (1994). J. Am. Chem. Soc..

[cit6] Fagan P. J., Burns E. G., Calabrese J. C. (1988). J. Am. Chem. Soc..

[cit7] (i) Wakamiya A. in Main Group Strategies towards Functional Hybrid Materials ed. T. Baumgartner and F. Jäkle, John Wiley & Sons Ltd., Hoboken (New Jersey), 2018, vol. 1, pp. 1–26

[cit8] Gross U., Kaufmann D. (1987). Chem. Ber..

[cit9] Su X., Bartholome T. A., Tidwell J. R., Pujol A., Yruegas S., Martinez J. J., Martin C. D. (2021). Chem. Rev..

[cit10] Saito M., Akiba T., Kaneko M., Kawamura T., Abe M., Hada M., Minoura M. (2013). Chem. – Eur. J..

[cit11] Braunschweig H., Hörl C., Hupp F., Radacki K., Wahler J. (2012). Organometallics.

[cit12] Heitkemper T., Naß L., Sindlinger C. P. (2020). Dalton Trans..

[cit13] Eisch J. J., Kaska W. C. (1962). J. Am. Chem. Soc..

[cit14] Drescher R., Lin S., Hofmann A., Lenczyk C., Kachel S., Krummenacher I., Lin Z., Braunschweig H. (2020). Chem. Sci..

[cit15] Drescher R., Ritschel B., Dewhurst R. D., Deißenberger A., Hofmann A., Braunschweig H. (2021). Chem. Commun..

[cit16] Klosin J., Roof G. R., Chen E. Y.-X., Abboud K. A. (2000). Organometallics.

[cit17] Haubold W., Herdtle J., Gollinger W., Einholz W. (1986). J. Organomet. Chem..

[cit18] Braunschweig H., Ye Q., Radacki K. (2012). Chem. Commun..

[cit19] Lavallo V., Canac Y., Präsang C., Donnadieu B., Bertrand G. (2005). Angew. Chem., Int. Ed..

[cit20] Braunschweig H., Dyakonov V., Jimenez-Halla J. O. C., Kraft K., Krummenacher I., Radacki K., Sperlich A., Wahler J. (2012). Angew. Chem., Int. Ed..

[cit21] Braunschweig H., Fernández I., Frenking G., Kupfer T. (2008). Angew. Chem., Int. Ed..

[cit22] Zhang Z., Edkins R. M., Haehnel M., Wehner M., Eichhorn A., Mailänder L., Meier M., Brand J., Brede F., Müller-Buschbaum K., Braunschweig H., Marder T. B. (2015). Chem. Sci..

[cit23] Braunschweig H. and Krummenacher I. in Organic Redox Systems: Synthesis, Properties, and Applications, ed. T. Nishinaga, John Wiley & Sons, Hoboken (New Jersey), 2016, pp. 503–519

[cit24] Renk T., Ruf W., Siebert W. (1976). J. Organomet. Chem..

[cit25] Scheibitz M., Bolte M., Bats J. W., Lerner H.-W., Nowik I., Herber R. H., Krapp A., Lein M., Holthausen M. C., Wagner M. (2005). Chem. – Eur. J..

[cit26] Braunschweig H., Chiu C.-W., Gamon D., Gruß K., Hörl C., Kupfer T., Radacki K., Wahler J. (2013). Eur. J. Inorg. Chem..

[cit27] Schacht W., Kaufmann D. (1987). Chem. Ber..

[cit28] Ura Y., Li Y., Xi Z., Takahashi T. (1998). Tetrahedron Lett..

[cit29] Adamo C., Barone V. (1999). J. Chem. Phys..

[cit30] Foster J. P., Weinhold F. (1980). J. Am. Chem. Soc..

[cit31] von Ragué Schleyer P., Maerker C., Dransfeld A., Jiao H., van Eikema Hommes N. J. R. (1996). J. Am. Chem. Soc..

[cit32] Iida A., Yamaguchi S. (2011). J. Am. Chem. Soc..

