# Supplementary material for: Boroles from alumoles: accessing boroles with alkyl-substituted backbones via transtrielation
Source: Chem Sci. 2023 Jul 19;14(34):9010–5. doi: 10.1039/d3sc02668j (PMC10466280; doi:10.1039/d3sc02668j)
Supplement: SC-014-D3SC02668J-s001 [file SC-014-D3SC02668J-s001.pdf]

Electronic Supplementary Material (ESI) for Chemical Science.
This journal is © The Royal Society of Chemistry 2023

Supporting Information for

Boroles from alumoles: accessing boroles with alkyl-substituted backbones via transtrielation

Josina L. Bohlen,[a,b] Lukas Endres,[a,b] Regina Drescher,[a,b] Krzystof Radacki,[a,b] Maximilian Dietz,[a,b] Ivo Krummenacher,[a,b] Holger Braunschweig*[a,b]

[a] *Institute for Inorganic Chemistry, Julius-Maximilians-Universität Würzburg, Am Hubland, 97074 Würzburg, Germany.*

[b] *Institute for Sustainable Chemistry & Catalysis with Boron, Julius-Maximilians-Universität Würzburg, Am Hubland, 97074 Würzburg, Germany.*

Contents

Methods and materials 2

Synthetic procedures 4

NMR spectra of isolated compounds 10

UV-vis spectra 36

Cyclic voltammetry 37

X-ray crystallographic data 39

Computational details 41

References 51

Methods and materials

All manipulations were performed either under an atmosphere of dry argon or *in vacuo* using standard Schlenk line or glovebox techniques. Deuterated solvents were dried over molecular sieves and degassed by three freeze-pump-thaw cycles prior to use. All other solvents were distilled and degassed from appropriate drying agents. Both deuterated and non-deuterated solvents were stored under argon over activated 4 Å molecular sieves. Liquid-phase NMR spectra were acquired on a Bruker Avance 500 spectrometer (^{1}H: 500.1 MHz, ^{11}B: 160.5 MHz, ^{13}C: 1265.8 MHz) at room temperature if not stated otherwise. Chemical shifts (δ) are reported in ppm and internally referenced to the carbon nuclei (^{13}C{^{1}H}) or residual protons (^{1}H) of the solvent. Heteronuclei NMR spectra are referenced to external standards (^{11}B: $BF_3{\cdot}OEt_2$). Resonances are given as singlet (s), doublet (d), triplet (t), septet (sept) or multiplet (m). High-resolution mass spectrometry (HRMS) data were obtained from a Thermo Scientific Exactive Plus spectrometer. UV-vis spectra were measured on a METTLER TOLEDO UV-vis-Excellence UV5 spectrophotometer at room temperature. Cyclic voltammetry experiments were performed using a *Gamry Instruments* Reference 600 potentiostat. A standard three-electrode cell configuration was employed using a platinum disk working electrode, a platinum wire counter electrode, and a silver wire, separated by a *Vycor* tip, serving as the reference electrode. Formal redox potentials are referenced to the ferrocene/ferrocenium ($[Cp_2Fe]^{+/0}$) redox couple by using decamethylferrocene ($[Cp^*{}_2Fe]$; $E_{1/2} = -0.427$ V in THF)[1] as an internal standard. Tetra-*n*-butylammonium hexafluorophosphate ($[n\text{Bu}_4\text{N}][\text{PF}_6]$) was employed as the supporting electrolyte. Compensation for resistive losses (*iR* drop) was employed for all measurements. Unless stated otherwise, solvents and reagents were purchased from Sigma-Aldrich or Alfa Aesar. 1-(η^5-1,2,4-tris(*tert*-butyl)cyclopentadienyl)-2,3,4,5-tetraethylalumole (**1**),[2] $DurBBr_2$,[3] $DurBCl_2$,[3] $MesBBr_2$,[4] $MesBCl_2$,[5] (*o*-tolyl)BBr_2,[6] (*o*-tolyl)BCl_2,[5] $[(\eta^5\text{-}C_5H_5)Fe(\eta^5\text{-}C_5H_4BBr_2)]$ ($FcBBr_2$),[7] $PhBCl_2$[4], $PhBBr_2$[4] and $C_4Et_4Li_2$[8] were synthesized using literature procedures.

Numbering of the carbon atoms for the assignment of NMR shifts was done in accordance with Figure S1.

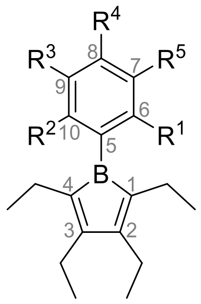

Figure S1. NMR numbering scheme for the borole compounds.

Synthetic procedures

Synthesis of $Me_2SnC_4Et_4$

To a solution of $C_4Et_4Li_2$ (250.0 mg, 1.40 mmol) in benzene (5 mL) was added dropwise a solution of Me_2SnCl_2 (308.2 mg, 1.40 mmol) in benzene (5 mL), leading to the immediate formation of a colorless solid (LiCl). The reaction mixture was stirred for 30 min at room temperature and then filtered. Evaporation of the solution at a pressure of 1.2 mbar afforded the product as a colorless oil (404.9 mg, 1.29 mmol, 92%). The spectral data match those reported in the literature.[9]

Synthesis of $MesBC_4Et_4$ (2)

A) To a solution of 1-(η^5-1,2,4-tris(*tert*-butyl)cyclopentadienyl)-2,3,4,5-tetraethylalumole (40.0 mg, 0.0944 mmol) in benzene (0.25 mL) was added dropwise a solution of dibromo(mesityl)borane (27.4 mg, 0.0944 mmol) in benzene (0.25 mL). The resulting reaction mixture was heated to 80 °C for 1 d and subsequently evaporated *in vacuo*. The residue was redissolved in pentane to remove the side product 1-(η^5-1,2,4-tris(*tert*-butyl)cyclopentadienyl)aluminum dibromide by addition of 1-(2,6-di*iso*propylphenyl)-3,3,5,5-tetramethylpyrrolidin-2-ylidene ($CAAC^{Me}$) and subsequent filtration. Evaporation of the remaining solution *in vacuo* afforded the product as a dark red oil (25.3 mg, 0.860 mmol, 91%).

B) To a solution of 2,3,4,5-tetraethyl-1,1-dimethyl-1*H*-stannole (250 mg, 0.799 mmol) in benzene (3 mL) was added dropwise a solution of dichloro(mesityl)borane (176 mg, 0.878 mmol) in benzene (2 mL). The resulting reaction mixture was heated to 100 °C for 7 d before all volatile components were removed *in vacuo*. Extraction of the residue with pentane and removal of the solvent *in vacuo* afforded a dark red oil (225 mg, 0.764 mmol, 96%).

^{1}H NMR (500 MHz, benzene-d_6): δ(ppm) = 6.77 (s, 2H, *m*-Ar*H*), 2.26 (s, 6H, (Ar-*o*-CH_3)$_2$), 2.19 (s, 3H, Ar-*p*-CH_3), 2.06 (q, 4H, C2(CH_2CH$_3$), C3(CH_2CH$_3$)), 2.04 (q, 4H, C1(CH_2CH$_3$), C4(CH_2CH$_3$)), 0.99 (t, ^3J = 7.58 Hz, 6H, C2(CH$_2$CH_3), C3(CH$_2$CH_3)), 0.88 (t, ^3J = 7.53 Hz, 6H, C1(CH$_2$CH_3), C4(CH$_2$CH_3)). **^{11}B NMR** (160 MHz, benzene-d_6): δ(ppm) = 77.58 (br s). **^{13}C NMR** (126 MHz, benzene-d_6): δ(ppm) = 164.1 (*C*2(CH$_2$CH$_3$), *C*3(CH$_2$CH$_3$)), 140.1 (*C*-*I*-Ar), 137.0 (*C*-*p*-Ar), 136.9 (br, *C*1(CH$_2$CH$_3$, *C*4(CH$_2$CH$_3$)), 135.8 (*C*-*o*-Ar), 127.4 (*C*H-Ar), 21.6 (*C*H$_3$-*o*-Ar), 21.3 (*C*H$_3$-*p*-Ar), 21.0 (C1(*C*H$_2$CH$_3$), C4(*C*H$_2$CH$_3$)), 20.2 (C2(*C*H$_2$CH$_3$), C3(*C*H$_2$CH$_3$)), 15.2 (C1(CH$_2$*C*H$_3$), C4(CH$_2$*C*H$_3$)), 14.2 ((C2(CH$_2$*C*H$_3$), C3(CH$_2$*C*H$_3$)). **HRMS LIFDI** for $[C_{21}H_{31}BH]^+$ = $[M+H^+]$: calcd. 295.2592; found 295.2579.

Synthesis of DurBC$_4$Et$_4$ (3)

A) To a solution of 1-(η^5-1,2,4-tris(*tert*-butyl)cyclopentadienyl)-2,3,4,5-tetraethylalumole (40 mg, 0.0944 mmol) in benzene (0.25 mL) was added dropwise a solution of dibromo(duryl)borane (28.7 mg, 0.0944 mmol) in benzene (0.25 mL). The resulting reaction mixture was heated to 80 °C for 3 d and evaporated *in vacuo*. The residue was redissolved in pentane to remove the side product 1-(η^5-1,2,4-tris(*tert*-butyl)cyclopentadienyl)aluminum dibromide by addition of 1-(2,6-di*iso*propylphenyl)-3,3,5,5-tetramethylpyrrolidin-2-ylidene (CAAC^{Me}) and subsequent filtration. Evaporation of the remaining solution *in vacuo* afforded a dark red oil (27.4 mg, 0.0856 mmol, 94%). Single crystals of **3** suitable for X-ray diffraction analysis were obtained by slow evaporation of a saturated hexane solution at –30 °C.

^{1}H NMR (500 MHz, benzene-d_6): δ(ppm) = 6.86 (s, 1H, *p*-Ar*H*), 2.17 (s, 6H, (Ar-*o*-CH_3)$_2$), 2.10 (s, 6H, (Ar-*m*-CH_3)$_2$)), 2.09 (m, 8H, C1(CH_2CH$_3$), C2(CH_2CH$_3$), C3(CH_2CH$_3$), C4(CH_2CH$_3$)), 1.01 (t, 6H, ^3J = 7.58 Hz, C1(CH$_2$CH_3), C4(CH$_2$CH_3)), 0.89 (t, 6H, ^3J = 7.53 Hz, C2(CH$_2$CH_3), C3(CH$_2$CH_3)). **^{11}B NMR** (160 MHz, benzene-d_6): δ(ppm) = 76.46 (br s). **^{13}C NMR** (126 MHz, benzene-d_6): δ(ppm) = 163.7 (*C*2(CH$_2$CH$_3$), *C*3(CH$_2$CH$_3$)), 143.4 (*C*-i-Ar), 137.1 ((*C*1(CH$_2$CH$_3$), *C*4(CH$_2$CH$_3$)), 133.3 (*C*-*o*-Ar), 131.3 (*C*-*m*-Ar), 131.1 (*C*H-Ar), 21.0 (C1(*C*H$_2$CH$_3$), (C4(*C*H$_2$CH$_3$)), 20.1 (C2(*C*H$_2$CH$_3$), (C3(*C*H$_2$CH$_3$)), 19.2 ((Ar-o-*C*H$_3$)$_2$), 19.0 ((Ar-*m*-*C*H$_3$)$_2$), 15.1 (C2(CH$_2$*C*H$_3$), C3(CH$_2$*C*H$_3$)), 14.2 (C1(CH$_2$*C*H$_3$), C4(CH$_2$*C*H$_3$)). **HRMS LIFDI** for [C$_{22}$H$_{33}$B] = [M]: calcd. 308.2675; found 308.2662.

Synthesis of FcBC$_4$Et$_4$ (4)

A) To a solution of 1-(η^5-1,2,4-tris(*tert*-butyl)cyclopentadienyl)-2,3,4,5-tetraethylalumole (20.0 mg, 0.0472 mmol) in pentane (0.25 mL) was added dropwise a solution of dibromo(ferrocenyl)borane (16.8 mg, 0.0472 mmol) in pentane (0.25 mL), upon which the reaction solution immediately turned dark orange. The side product 1-(η^5-1,2,4-tris(*tert*-butyl)cyclopentadienyl)aluminum dibromide was removed by addition of 1-(2,6-di*iso*propylphenyl)-3,3,5,5-tetramethylpyrrolidin-2-ylidene (CAAC^{Me}) and subsequent filtration. Evaporation of the remaining solution *in vacuo* afforded a dark red oil (12.4 mg, 0.0344 mmol, 73%).

B) To a solution of 2,3,4,5-tetraethyl-1,1-dimethyl-1*H*-stannole (40.0 mg, 0.128 mmol) in benzene (0.25 mL) was added dropwise a solution of dibromo(ferrocenyl)borane (45.4 mg, 0.878 mmol) in benzene (0.25 mL). All volatile components were removed *in vacuo*. Extraction

of the residue with pentane and removal of the solvent *in vacuo* afforded a dark orange oil (44.0 mg, 0.122 mmol, 96%).

^{1}H NMR (500 MHz, benzene-d_6): δ(ppm) = 4.54 (t, ^3J = 1.83 Hz, 2H, C7*H*, C8*H*), 4.51 (t, ^3J = 1.85 Hz, 2H, C6*H*, C9*H*), 3.98 (s, 5H, (Cp-C*H*), 2.33 (q, ^3J = 7.43 Hz, 4H, C1(CH_2CH$_3$), C4(CH_2CH$_3$)), 2.26 (q, ^3J = 7.56 Hz, 4H, C2(CH_2CH$_3$), C3(CH_2CH$_3$)), 1.18 (t, ^3J = 7.52 Hz, 6H, C1(CH$_2$CH_3), C4(CH$_2$CH_3)), 1.10 (t, ^3J = 7.56 Hz, 6H, C2(CH$_2$CH_3), C3(CH$_2$CH_3)). **^{11}B NMR** (160 MHz, benzene-d_6): δ(ppm) = 54.65 (br s). **^{13}C NMR** (126 MHz, benzene-d_6): δ(ppm) = 160.2 (*C*2(CH$_2$CH$_3$), *C*3(CH$_2$CH$_3$)), 138.0 (*C*1(CH$_2$CH$_3$), *C*4(CH$_2$CH$_3$)), 78.0 (*C*6, *C*9), 77.0 (*C*7, *C*8), 75.6 (*C*5), 70.7 (*C*H-Cp), 21.8 (C1(*C*H$_2$CH$_3$), C4(*C*H$_2$CH$_3$)), 20.0 (C2(*C*H$_2$CH$_3$), C3(*C*H$_2$CH$_3$)), 16.6 (C1(CH$_2$*C*H$_3$), C4(CH$_2$*C*H$_3$)), 15.2 (C2(CH$_2$*C*H$_3$), C3(CH$_2$*C*H$_3$)). **HRMS LIFDI** for $[C_{22}H_{29}BFe]^+$ = [M+H$^+$]: calcd. 360.1706; found 360.1688.

Synthesis of PhBC$_4$Et$_4$ (5)

To a solution of 1-(η^5-1,2,4-tris(*tert*-butyl)cyclopentadienyl)-2,3,4,5-tetraethylalumole (20.0 mg, 0.047 mmol) in benzene (0.25 mL) was added dropwise a solution of dibromo(phenyl)borane (11.7 mg, 0.047 mmol) in benzene (0.25 mL), upon which the reaction solution quickly turned red with almost immediate discoloration. Removal of all volatile components *in vacuo* afforded a colorless oil. The oil was redissolved in pentane. The side product 1-(η^5-1,2,4-tris(*tert*-butyl)cyclopentadienyl)aluminum dibromide could not be removed by addition of 1-(2,6-di*iso*propylphenyl)-3,3,5,5-tetramethylpyrrolidin-2-ylidene (CAAC^{Me}) without partial formation of the respective adduct **5a**.

Synthesis of (CAAC^{Me})PhBC$_4$Et$_4$ (5a)

To a solution of 1-(η^5-1,2,4-tris(*tert*-butyl)cyclopentadienyl)-2,3,4,5-tetraethylalumole (20.0 mg, 0.047 mmol) in benzene (0.25 mL) was added dropwise a solution of dibromo(phenyl)borane (11.7 mg, 0.047 mmol) in benzene (0.25 mL), upon which the reaction solution quickly turned red with almost immediate discoloration. All volatile components were removed *in vacuo*. The residue was redissolved in pentane to remove the side product 1-(η^5-1,2,4-tris(*tert*-butyl)cyclopentadienyl)aluminum dibromide by addition of 1-(2,6-di*iso*propylphenyl)-3,3,5,5-tetramethylpyrrolidin-2-ylidene (CAAC^{Me}) and subsequent filtration. The addition of another equivalent of CAAC^{Me} led to a change in color from colorless to intensively orange alongside with the formation of an orange solid. The removal of all volatiles *in vacuo* and subsequent washing with pentane resulted in the isolation of a crystalline

orange solid (11.2 mg, 0.0472 mmol, 44%). Single crystals of **5a** suitable for X-ray diffraction analysis were obtained by slow evaporation of a saturated pentane solution at 20 °C.

^{1}H NMR (500 MHz, benzene-*d$_6$*): δ(ppm) = 7.13 (t, ^3J = 7.77 Hz, 1H, dip-*p*-C*H*), 6.96–6.91 (m, 5H, dip-*m*-C*H*, ph-*o*-C*H*, ph-*p*-C*H*), 6.85–6.81 (m, 2H, ph-*m*-C*H*), 3.04 (sept, ^3J = 6.63 Hz, 2H, C(C*H*(CH$_3$)$_2$), 2.72–2.64 (m, 2H, C1(C*H*$_2$CH$_3$) and C4(C*H*$_2$CH$_3$); one hydrogen atom of CH$_2$, respectively), 2.61–2.48 (m, 6H, C1(C*H*$_2$CH$_3$) and C4(C*H*$_2$CH$_3$); one hydrogen atom of CH$_2$, respectively and C2(C*H*$_2$CH$_3$), C3(C*H*$_2$CH$_3$)), 1.62 (s, 6H, C$_{carbene}$C(C*H*$_3$)$_2$), 1.46 (s, 2H, C*H*$_2$), 1.35 (t, ^3J = 7.53 Hz, 6H, C2(CH$_2$C*H*$_3$), C3(CH$_2$C*H*$_3$)), 1.12 (d, ^3J = 6.70 Hz, 6H, C(CH(C*H*$_3$)$_2$)), 1.08–1.06 (m, 12H, C(CH(C*H*$_3$)$_2$) and C1(CH$_2$C*H*$_3$), C4(CH$_2$C*H*$_3$)), 0.98–0.95 (m, 12H, NC(C*H*$_3$)$_2$). **^{11}B NMR** (160 MHz, benzene-*d$_6$*): δ(ppm) = –3.90. **^{13}C NMR** (126 MHz, benzene-*d$_6$*): δ(ppm) = 241.5 (*C*$_{carbene}$), 160.2 (C1, C4), 150.4 (C5), 150.1 (C2, C3), 146.0 (*C*-*o*-dip), 138.2 (*C*-*i*-dip), 134.8 (*C*-*o*-ph), 129.9 (*C*-*p*-dip), 125.9 (*C*-*m*-ph), 125.6 (*C*-*m*-dip), 122.9 (*C*-*p*-ph), 76.9 (N*C*(CH$_3$)$_2$), 57.1 (C$_{carbene}$*C*(CH$_3$)$_2$), 53.5 (*C*H$_2$), 34.8 (C$_{carbene}$C(*C*H$_3$)$_2$), 29.4 (NC(*C*H$_3$)$_2$), 29.0 (C(*C*H(CH$_3$)$_2$), 25.9 (C1(*C*H$_2$CH$_3$), C4(*C*H$_2$CH$_3$)), 25.8 (C(CH(*C*H$_3$)$_2$), 25.3 (C(CH(*C*H$_3$)$_2$), 21.2 (C2(*C*H$_2$CH$_3$), C3(*C*H$_2$CH$_3$)), 16.3 (C1(CH$_2$*C*H$_3$), C4(CH$_2$*C*H$_3$)), 15.2 (C2(CH$_2$*C*H$_3$), C3(CH$_2$C*H*$_3$). **HRMS LIFDI** for [$C_{38}H_{56}BN$] = [M]: calcd. 537.4500; found 538.4490. **Elemental analysis** calcd. for $C_{38}H_{56}BN$ [537.68 g/mol]: C 84.89, H 10.50, N 2.61%; found: C 82.57, H 10.32, N 2.62%.

Synthesis of *o*-TolBC$_4$Et$_4$ (6)

A) To a solution of 1-(η^5-1,2,4-tris(*tert*-butyl)cyclopentadienyl)-2,3,4,5-tetraethylalumole (22.0 mg, 0.0519 mmol) in benzene (0.25 mL) was added dropwise a solution of dichloro(*o*-tolyl)borane (13.6 mg, 0.0519 mmol) in benzene (0.25 mL), upon which the reaction solution immediately turned intensively purple. Removal of all volatile components *in vacuo* afforded a dark purple oil. The residue was redissolved in pentane. The side product 1-(η^5-1,2,4-tris(*tert*-butyl)cyclopentadienyl)aluminum dibromide could not be removed by addition of 1-(2,6-di*iso*propylphenyl)-3,3,5,5-tetramethylpyrrolidin-2-ylidene (CAAC^{Me}) and subsequent filtration without partial formation of the respective adduct **6a**.

B) To a solution of 2,3,4,5-tetraethyl-1,1-dimethyl-1*H*-stannole (40.0 mg, 0.128 mmol) in benzene (0.25 mL) a solution of dichloro(*o*-tolyl)borane (23.0 mg, 0.133 mmol) was added dropwise, upon which the reaction solution immediately turned intensively purple. All volatile components were removed *in vacuo*. Extraction of the residue with pentane and removal of the solvent *in vacuo* afforded a dark purple oil (31.0 mg, 0.116 mmol, 91%).

^{1}**H NMR** (500 MHz, benzene-d_6, 343 K): δ(ppm)= 7.21–7.19 (m, 1H, *o*-Ar*H*), 7.14–7.11 (m, 2H, C8*H*, C9*H*), 7.10–7.05 (m, 1H, C7*H*), 2.30 (s, 3H, Ar-*o*-CH_3), 2.09 (q, ^{3}J = 7.62 Hz, 4H, C2(CH_2CH$_3$), C3(CH_2,CH$_3$)), 2.05 (q, ^{3}J = 7.77 Hz, 4H, C1(CH_2CH$_3$), C4(CH_2,CH$_3$)), 1.01 (t, ^{3}J = 7.57 Hz, 6H, C2(CH$_2$CH_3), C3(CH$_2$CH_3)), 0.83 (t, ^{3}J = 7.50 Hz, 6H, C1(CH$_2$CH_3), C4(CH$_2$CH_3)). ^{11}**B NMR** (160 MHz, benzene-d_6, 343 K): δ(ppm)= 74.57 (br s). ^{13}**C NMR** (126 MHz, benzene-d_6, 343 K): δ(ppm)= 164.6 (*C*2(CH$_2$CH$_3$), *C*3(CH$_2$CH$_3$)), 143.6 (C5), 137.8 (C6), 137.8 (br.; (*C*1(CH$_2$CH$_3$), *C*4(CH$_2$CH$_3$)), 129.3 (C7), 128.4 (C8), 127.4 (C10), 125.1 (C9), 22.5 (*C*H$_3$-*o*-Ar), 20.6 (C2(*C*H$_2$CH$_3$), C3(*C*H$_2$CH$_3$)), 20.2 (C1(*C*H$_2$CH$_3$, C4(*C*H$_2$CH$_3$)), 15.0 (C1(CH$_2$*C*H$_3$, C4(CH$_2$*C*H$_3$)), 13.9 (C2(CH$_2$*C*H$_3$, C3(CH$_2$*C*H$_3$)).
HRMS LIFDI for $[C_{19}H_{27}B]^+$ = [M$^+$]: calcd. 266.2200; found 266.2195.

Synthesis of (CAAC^{Me})*o*-TolBC$_4$Et$_4$ (6a)

To a solution of 1-(η^5-1,2,4-tris(*tert*-butyl)cyclopentadienyl)-2,3,4,5-tetraethylalumole (50.0 mg, 0.118 mmol) in benzene (0.25 mL) was added dropwise a solution of dibromo(*o*-tolyl)borane (20.4 mg, 0.118 mmol) in benzene (0.25 mL), upon which the reaction solution immediately turned intensively purple. All volatile components were removed *in vacuo.* The residue was redissolved in pentane to remove the side product 1-(η^5-1,2,4-tris(tert-butyl)cyclopentadienyl)aluminum dibromide by addition of 1-(2,6-di*iso*propylphenyl)-3,3,5,5-tetramethylpyrrolidin-2-ylidene (CAAC^{Me}) and subsequent filtration. The addition of another equivalent of CAAC^{Me} led to the formation of a red solid. The removal of all volatiles *in vacuo* and subsequent washing with pentane resulted in the isolation of a crystalline orange-red solid (20.50 mg, 0.0372 mmol, 33%). Single crystals of **6a** suitable for X-ray diffraction analysis were obtained by slow evaporation of a saturated hexane solution at –30 °C.

^{1}**H NMR** (500 MHz, benzene-d_6): δ(ppm) = 7.06 (t, ^{3}J = 7.75 Hz, 1H, dip-*p*-C*H*), 6.94 (d, ^{3}J = 7.30 Hz, 1H, C7*H*), 6.89 (d, ^{3}J = 7.75 Hz 2H, dip-*m*-C*H*), 6.82 (apparent dt, ^{3}J = 7.30 Hz, 1H, C8*H*), 6.80 (d, ^{3}J = 7.55 Hz, 1H, C10*H*), 6.28–6.25 (m, 1H, C19*H*), 3.08 (sept, ^{3}J = 6.60 Hz, 2H, C(C*H*(CH$_3$)$_2$), 2.71–2.64 (m, 2H, C1(CH_2CH$_3$) and C4(CH_2CH$_3$); one hydrogen atom of CH$_2$, respectively), 2.59–2.49 (m, 6H, C1(CH_2CH$_3$) and C4(CH_2CH$_3$); one hydrogen atom of CH$_2$, respectively and C2(CH_2CH$_3$), C3(CH_2CH$_3$)), 2.39 (s, 3H, CH$_3$-*o*-Ar), 1.62 (s, 6H, $C_{carbene}$C(CH_3)$_2$), 1.47 (s, 2H, CH_2), 1.35 (t, ^{3}J = 7.50 Hz, 6H, C2(CH$_2$CH_3), C3(CH$_2$CH_3)), 1.17 (d, ^{3}J = 6.55 Hz, 6H, C(CH(CH_3)$_2$)), 1.08 (d, ^{3}J = 6.60 Hz, 6H, C(CH(CH_3)$_2$)), 0.98–0.95 (m, 12H, NC(CH_3)$_2$ and C1(CH$_2$CH_3), C4(CH$_2$CH_3)). ^{11}**B NMR** (160 MHz, benzene-d_6): δ(ppm) = –2.67. ^{13}**C NMR** (126 MHz, benzene-d_6): δ(ppm) = 242.2

(*C*$_{carbene}$), 157.2 (C1, C4), 156,8 (C1, C4), 151.5 (C2, C3), 146.3 (C5), 145.4 (*C*-*o*-dip), 141.6 (C6), 139.0 (C10), 138.8 (*C*-*i*-dip), 130.0 (C7), 129.7 (*C*-*p*-dip), 125.6 (*C*-*m*-dip), 122.3 (C8), 121.2 (C9), 77.6 (N*C*(CH_3)$_2$), 58.3 (C$_{carbene}$*C*(CH_3)$_2$), 53.0 (*C*H_2), 34.8 (C$_{carbene}$C(*C*H_3)$_2$), 29.7 (NC(*C*H_3)$_2$), 28.7 (C(*C*H(CH_3)$_2$), 26.3 (C1(*C*$H_2$$CH_3$), C4(*C*$H_2$$CH_3$)), 26.1 (C(CH(*C*$H_3$)$_2$), 24.9 (C(CH(*C*$H_3$)$_2$), 23.7 (*C*$H_3$-*o*-Ar), 21.3 (C2(*C*$H_2$$CH_3$), C3(*C*$H_2$$CH_3$)), 16.2 (C1($CH_2$*C*$H_3$), C4($CH_2$*C*$H_3$)), 14.7 (C2($CH_2$*C*$H_3$), C3($CH_2$C*H*$_3$). **HRMS LIFDI** for [$C_{39}H_{58}BN$] = [M]: calcd. 551.4657; found 551.4648. **Elemental analysis** calcd. for $C_{39}H_{58}BN$ [551.71 g/mol]: C 84.90, H 10.60, N 2.54%; found: C 83.90, H 10.57, N 2.47%.

NMR spectra of isolated compounds

Note: Additional small signals are observed in the NMR spectra of the boroles, indicating the presence of residual side products that were not completely removed during the workup of the reactions. These signals correspond to either aluminum dibromide or dimethyltin dihalide species. The captions of the NMR spectra also indicate the route (Al or Sn) by which the products were obtained.

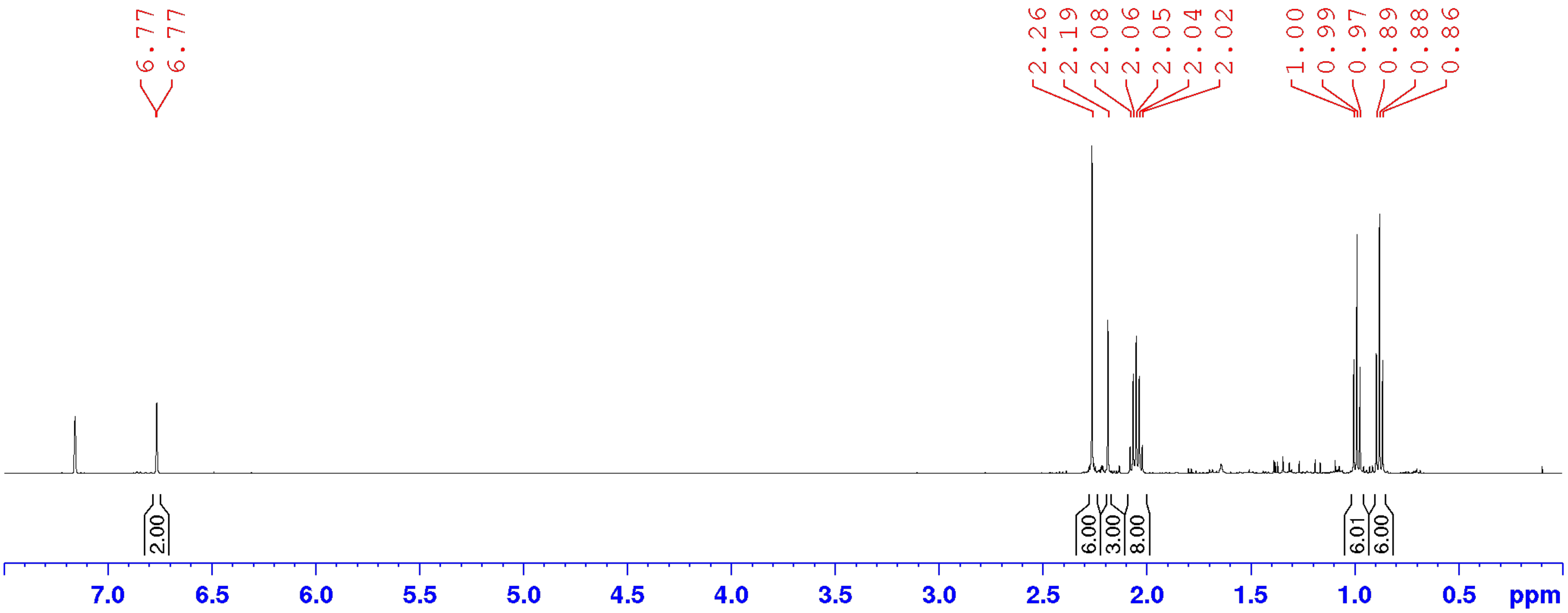

Figure S2. $^{1}H\{^{11}B\}$ NMR spectrum of **2** in C_6D_6 (Al route).

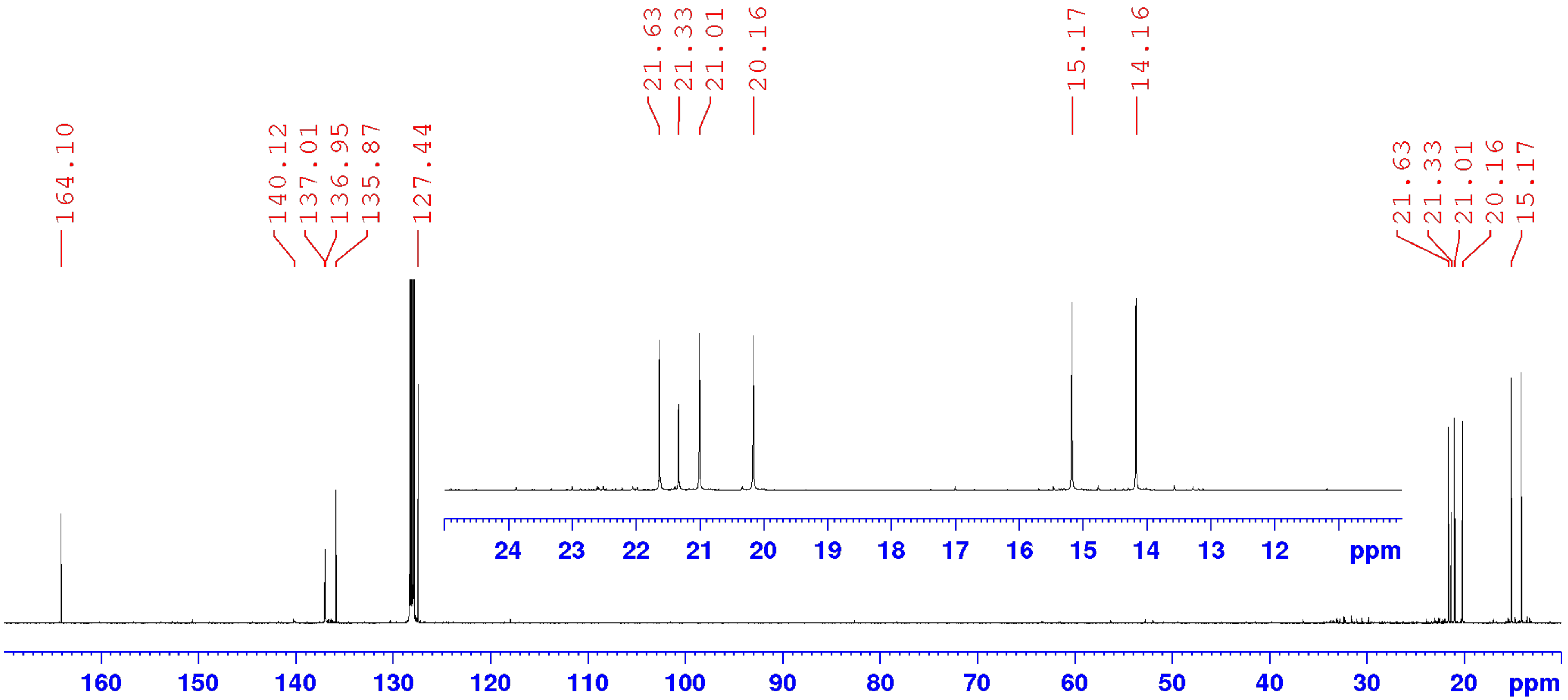

Figure S3. $^{13}C\{^1H\}$ NMR spectrum of **2** in C_6D_6 (Al route).

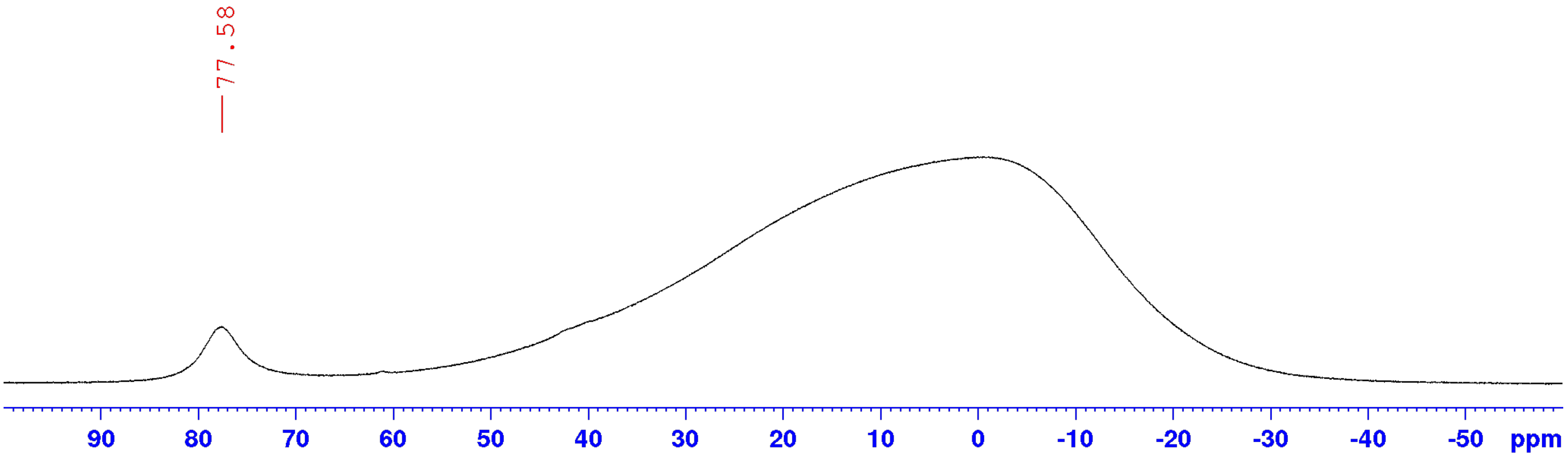

Figure S4. ^{11}B NMR spectrum of **2** in C_6D_6 (Al route).

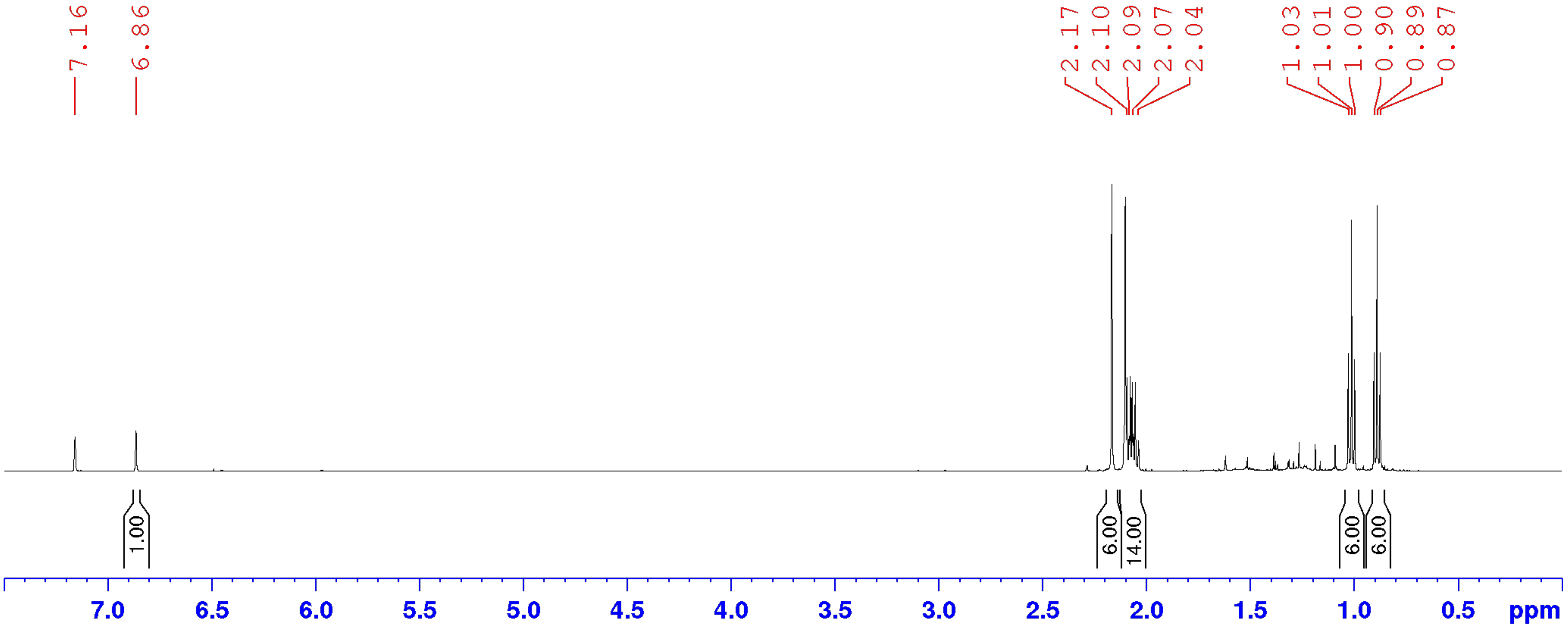

Figure S5. $^{1}H\{^{11}B\}$ NMR spectrum of **3** in C_6D_6 (Al route).

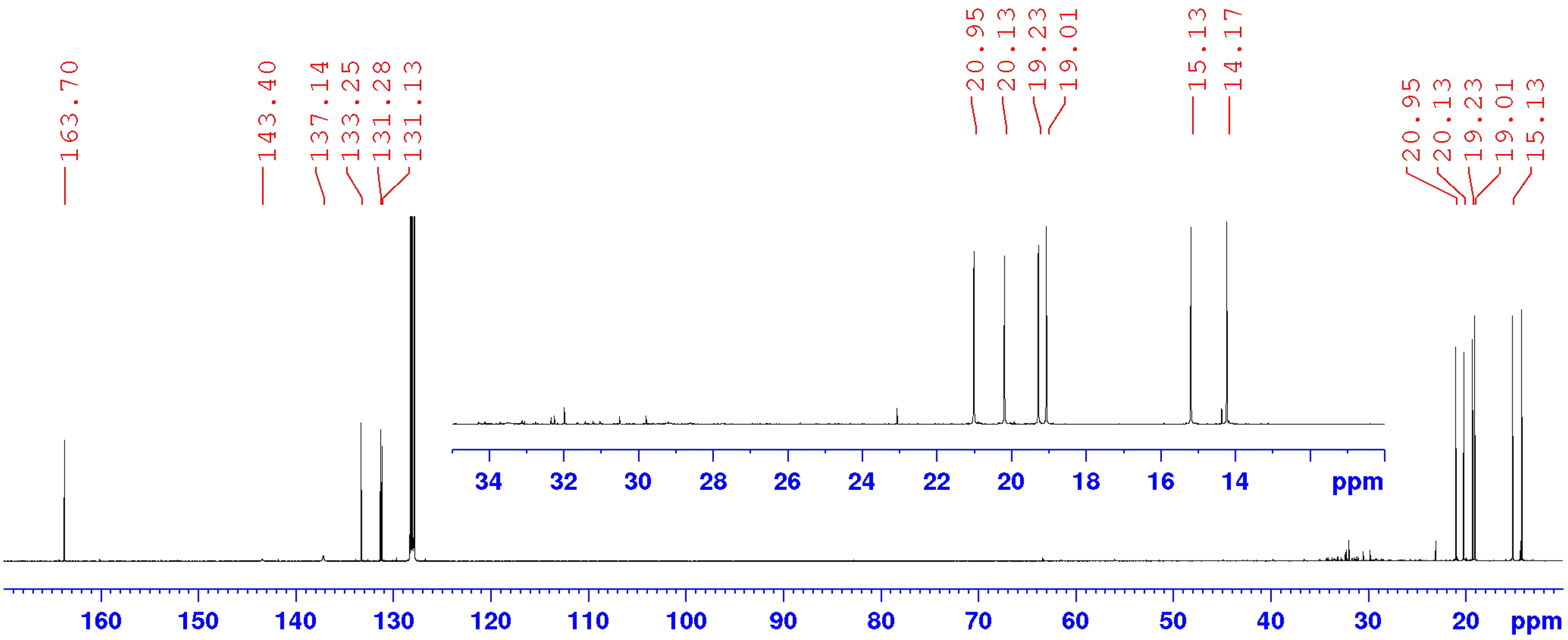

Figure S6. $^{13}C\{^{1}H\}$ NMR spectrum of **3** in C_6D_6 (Al route).

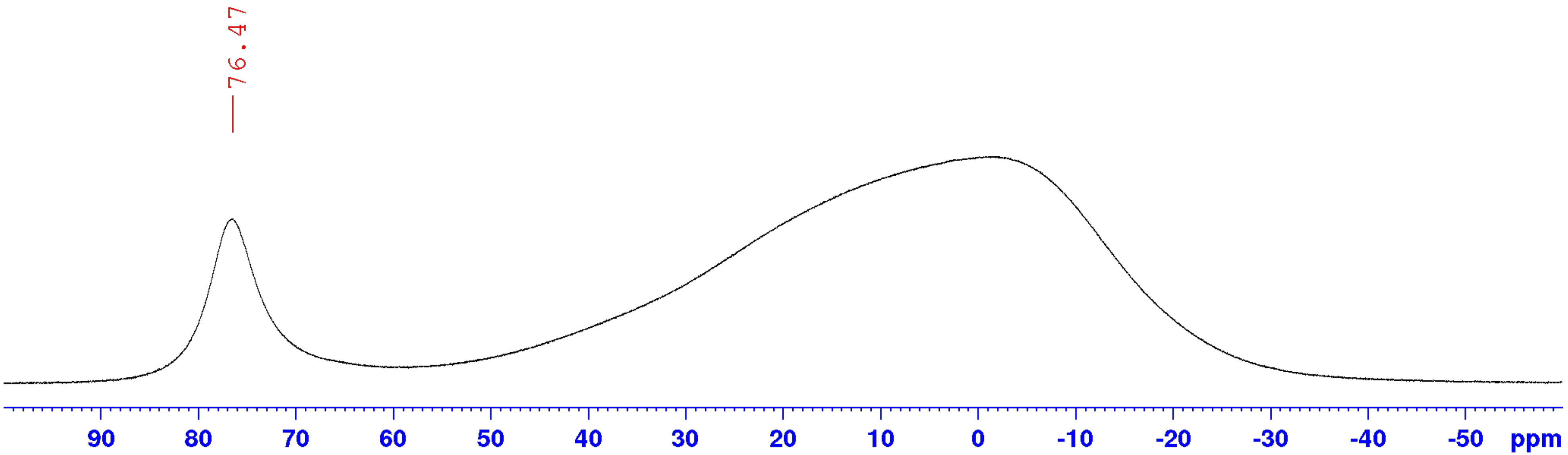

Figure S7. ^{11}B NMR spectrum of **3** in C_6D_6 (Al route).

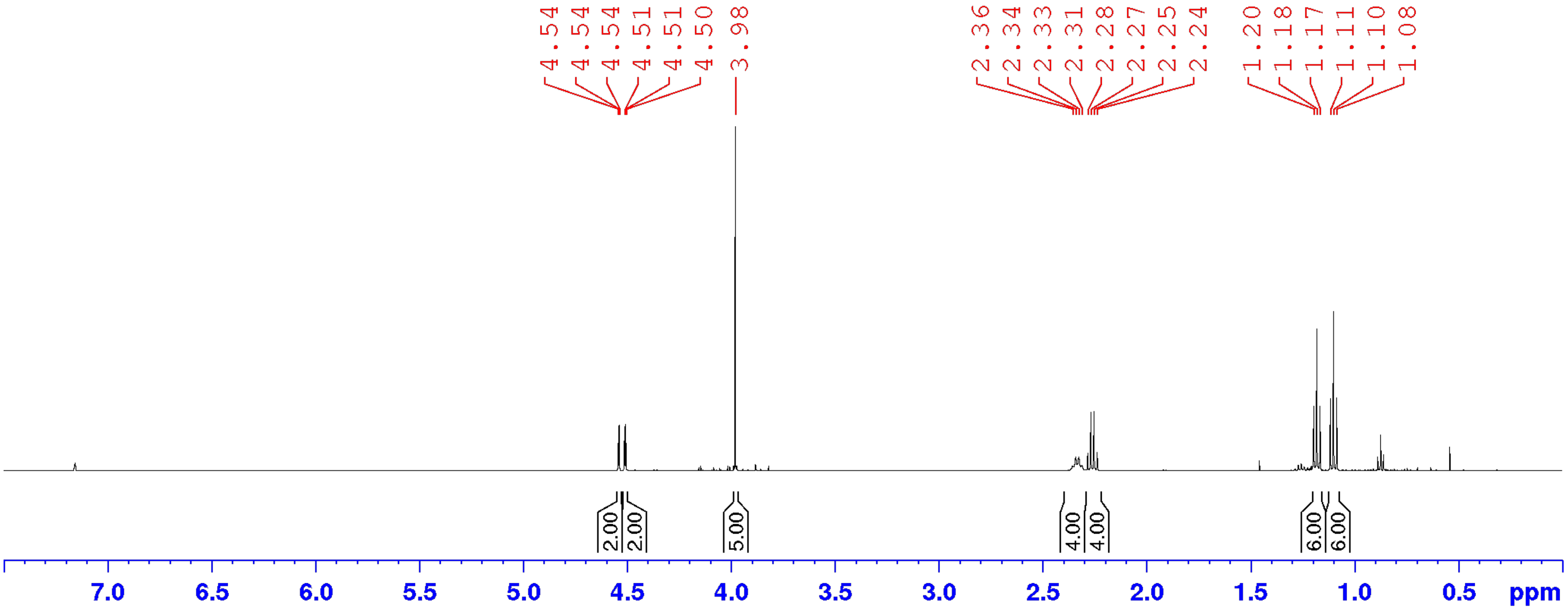

Figure S8. $^{1}H\{^{11}B\}$ NMR spectrum of **4** in C_6D_6 (Sn route).

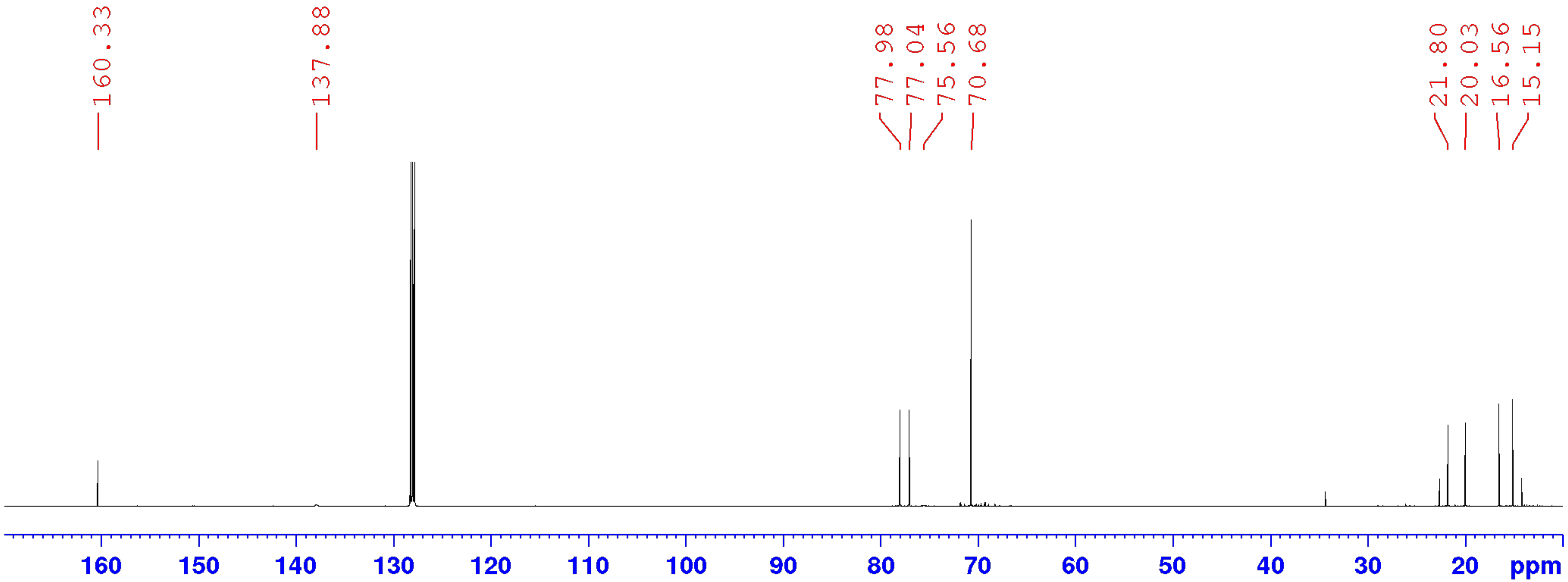

Figure S9. $^{13}C\{^{1}H\}$ NMR spectrum of **4** in C_6D_6 (Sn route).

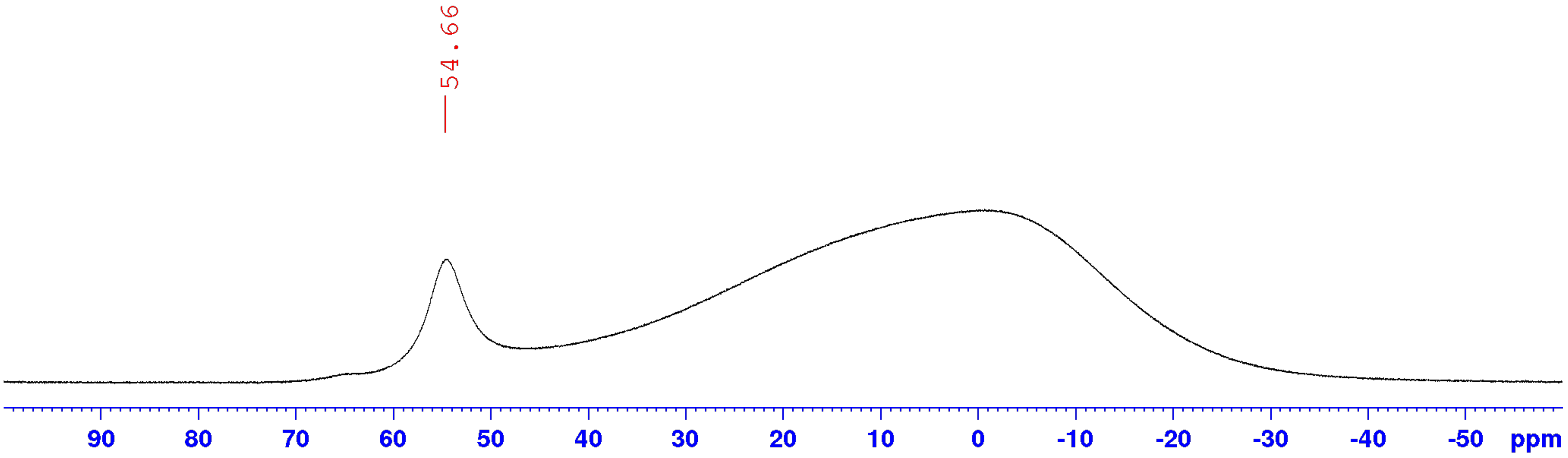

Figure S10. ^{11}B NMR spectrum of **4** in C_6D_6 (Sn route).

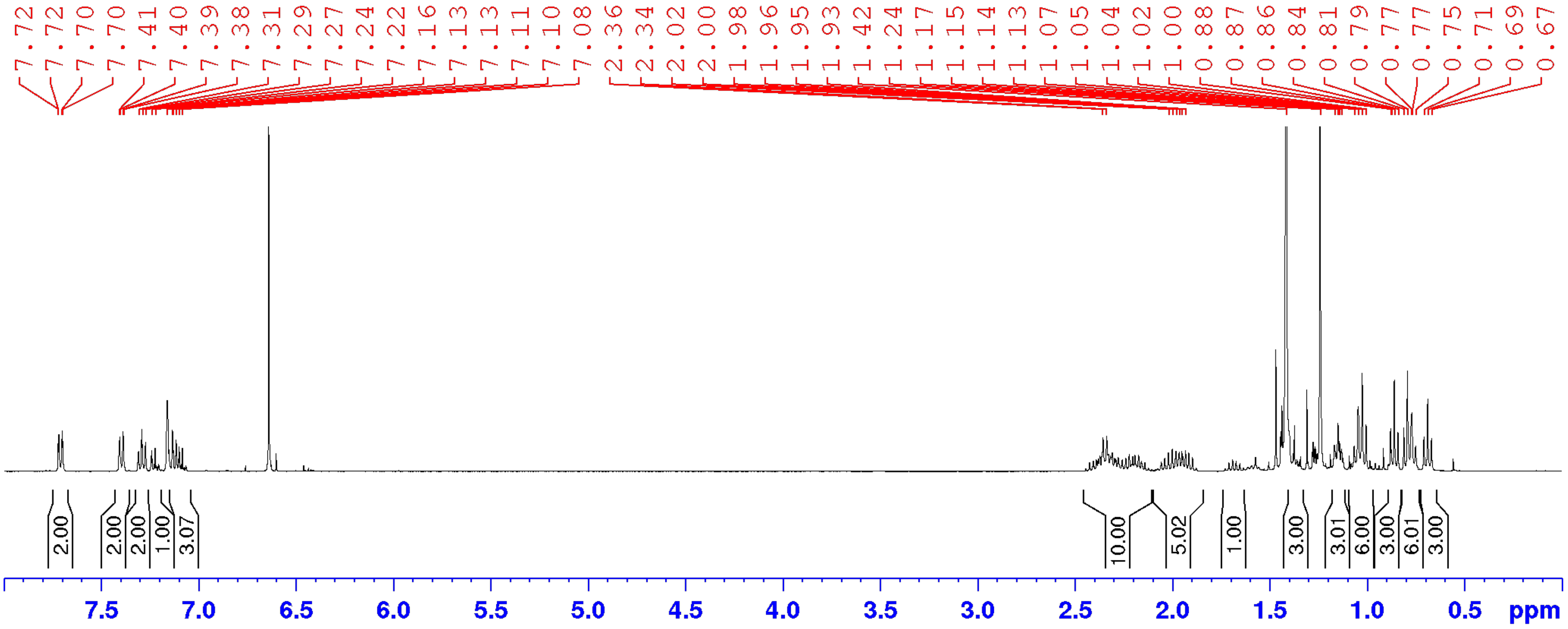

Figure S11. ^{1}H NMR spectrum of **5** in C_6D_6. The peaks at δ(ppm) = 6.64 (s, 2H, Ar*H*), 1.42 (s, 18 H, $C(CH_3)_2(CH_3)$), and 1.24 (s, 9H, $C(CH_3)_2(CH_3)$) are due to the side product 1-(η^5-1,2,4-tris(*tert*-butyl)cyclopentadienyl)aluminum dibromide (Al route).

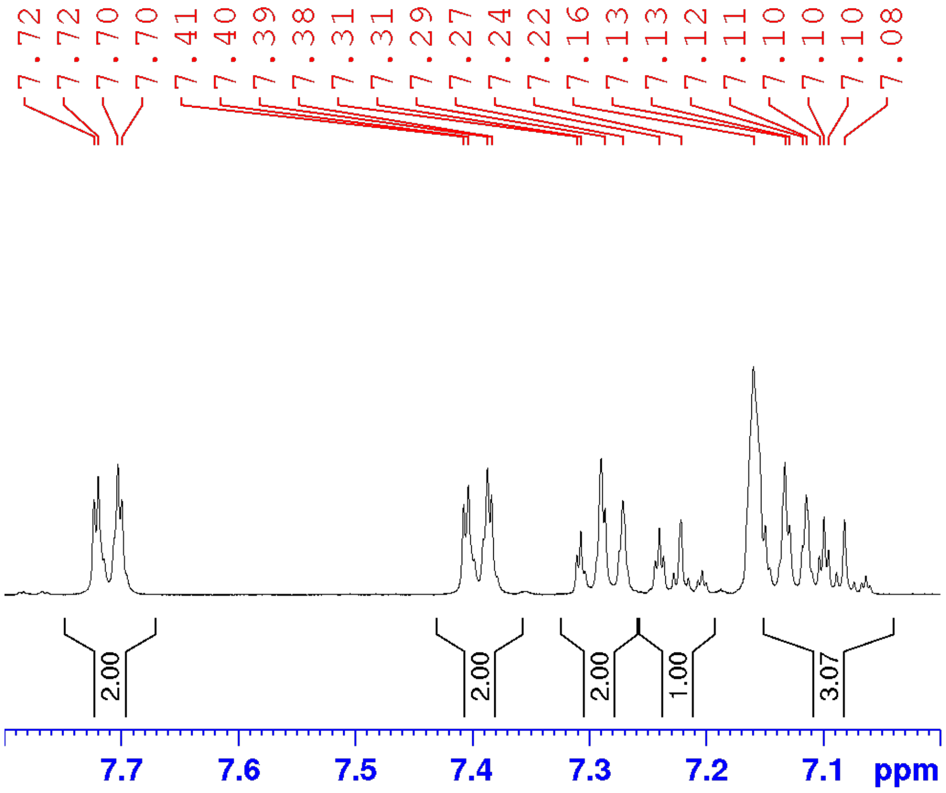

Figure S12. ^1H NMR spectrum of **5** in C_6D_6 with expansion of the aryl region (Al route).

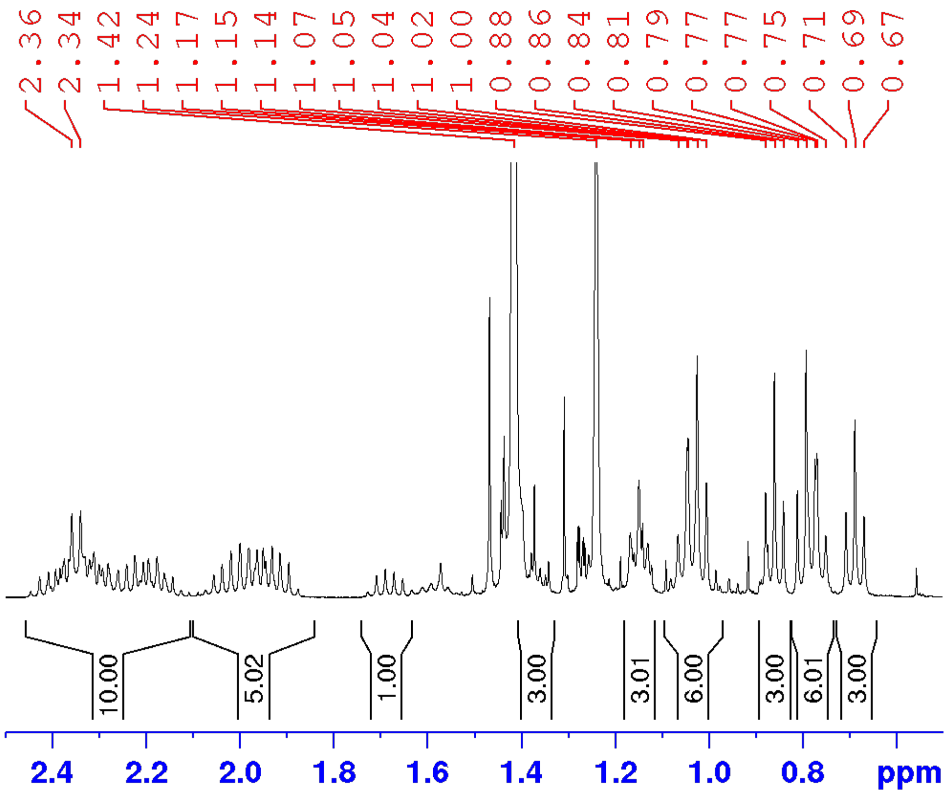

Figure S13. ^1H NMR spectrum of **5** in C_6D_6 with expansion of the aliphatic region (Al route).

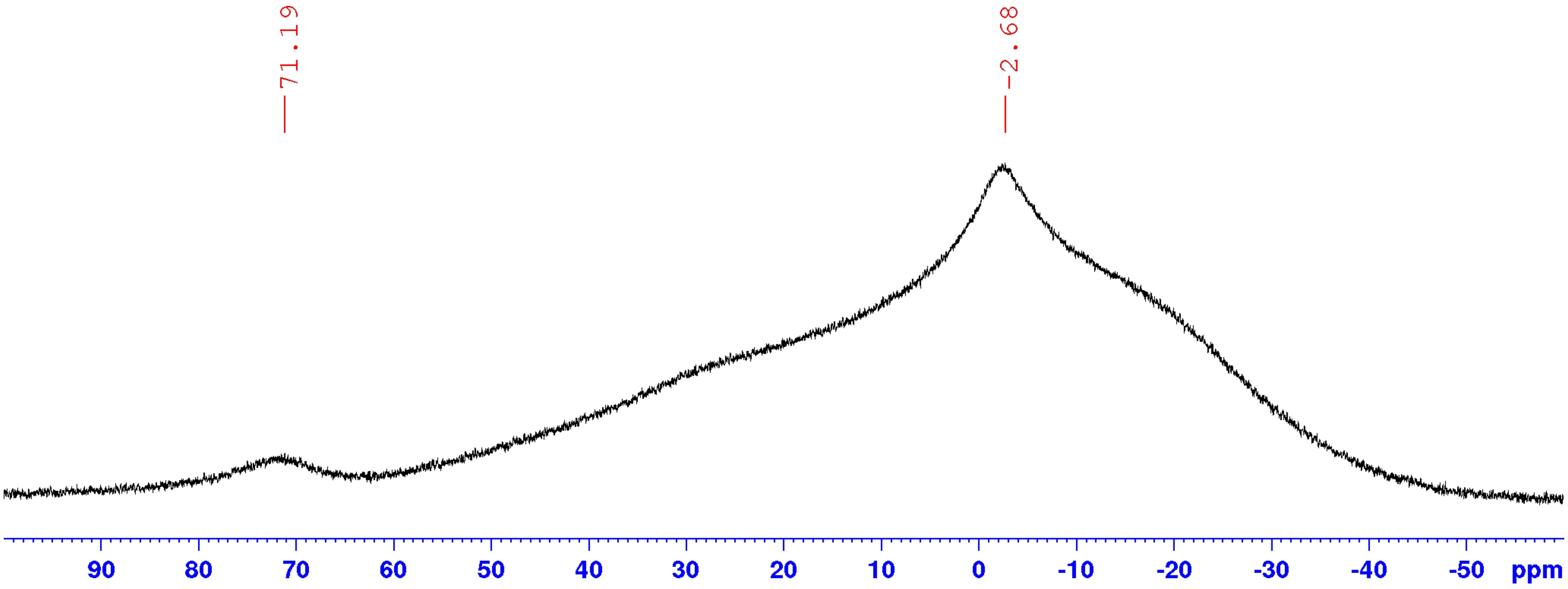

Figure S14. ^{11}B NMR spectrum of **5** in C_6D_6 (Al route).

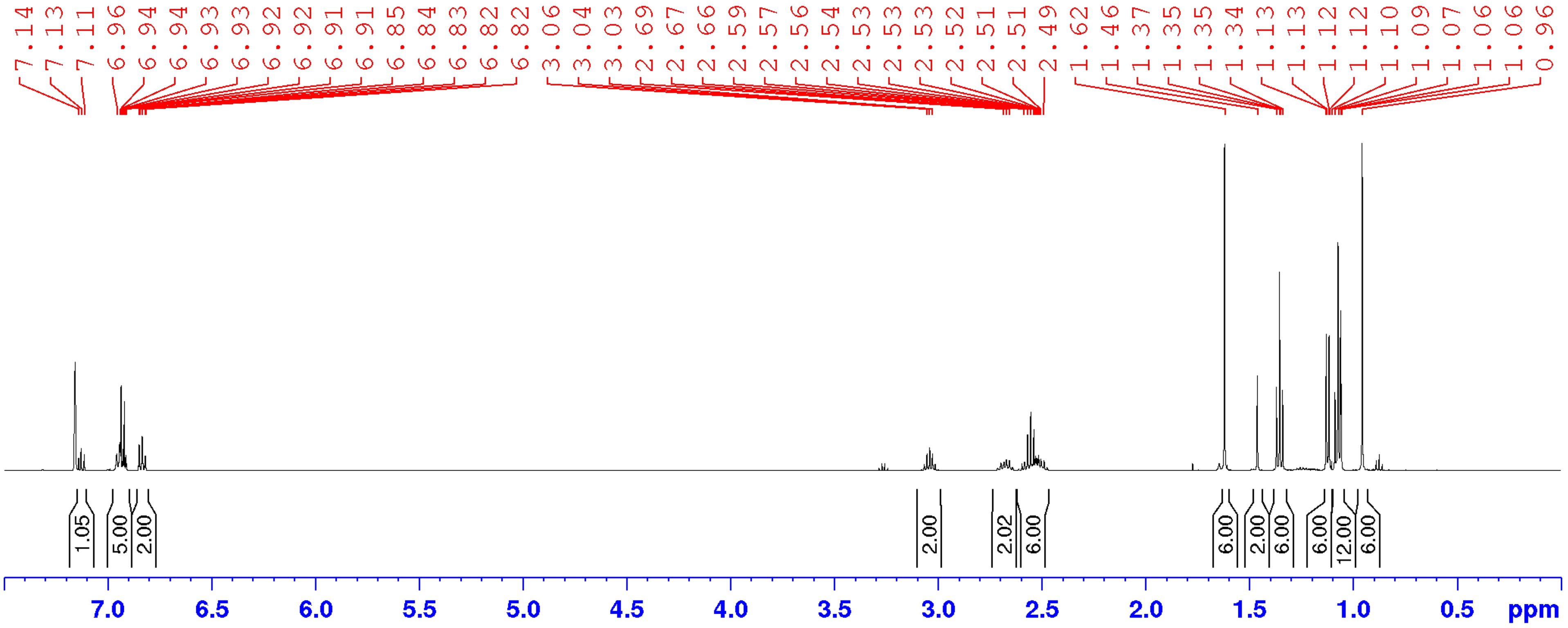

Figure S15. ^{1}H NMR spectrum of **5a** in C_6D_6.

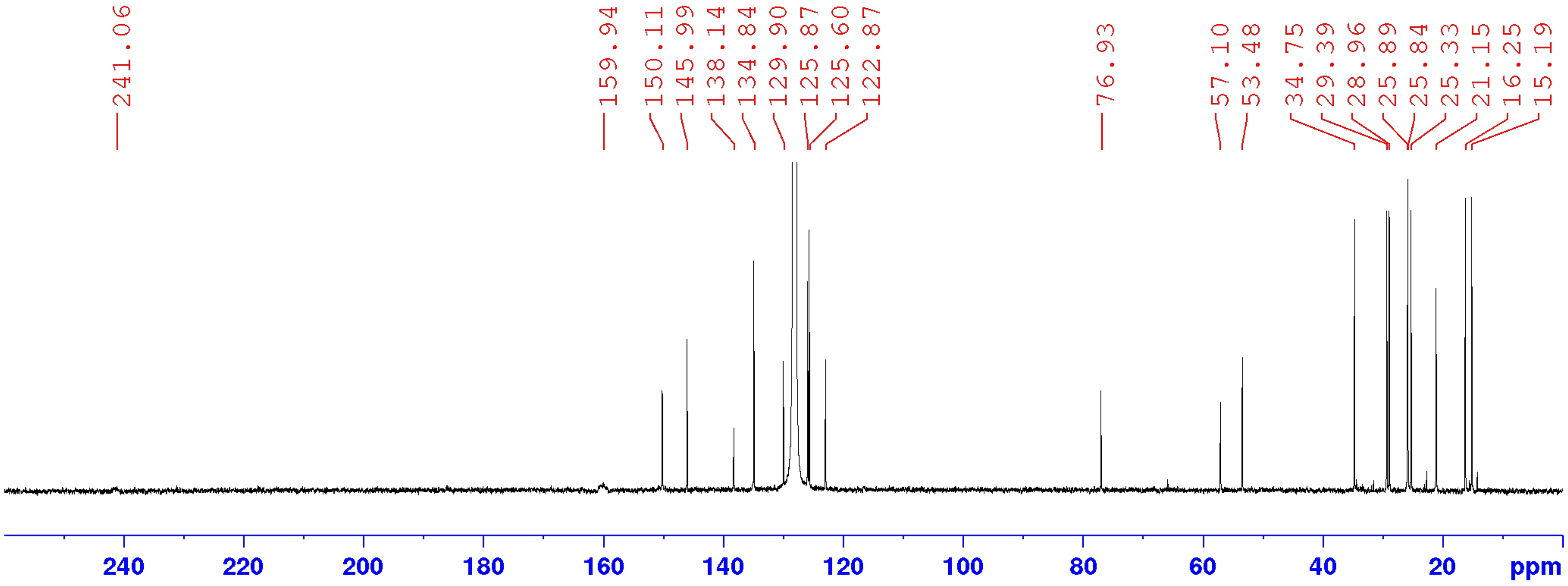

Figure S16. $^{13}C\{^{1}H\}$ NMR spectrum of **5a** in C_6D_6.

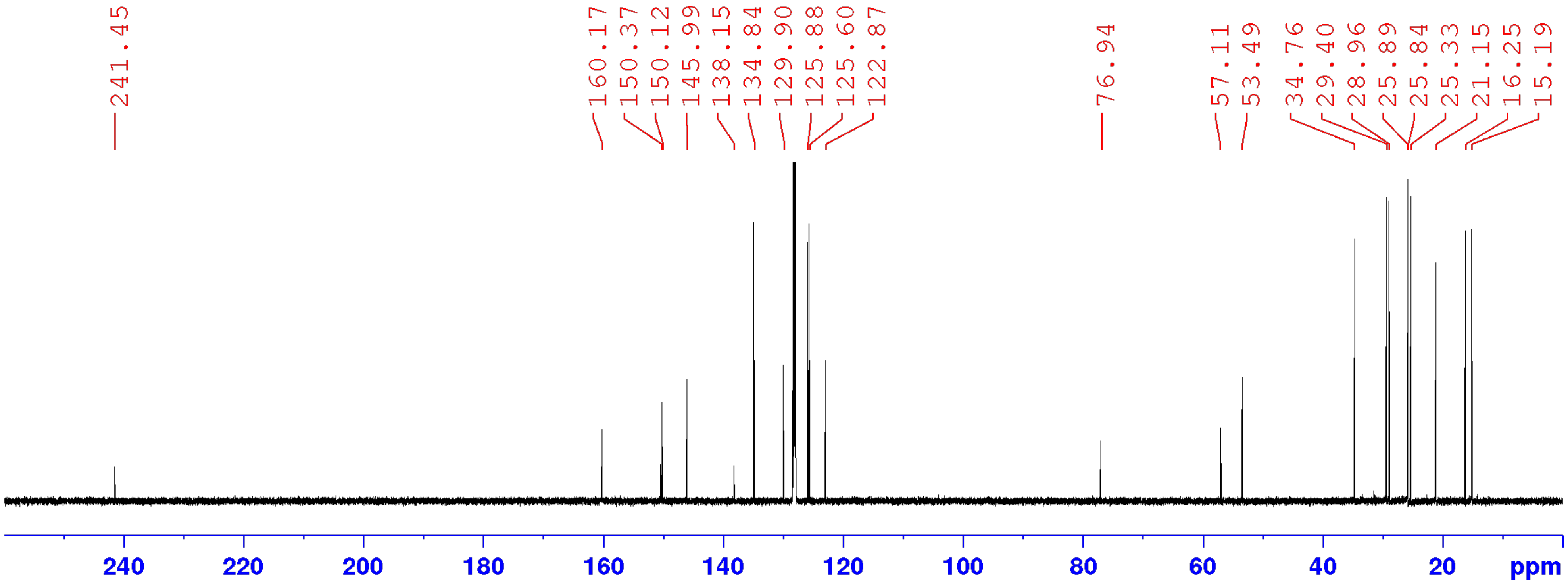

Figure S17. $^{13}C\{^{1}H,^{11}B\}$ NMR spectrum of **5a** in C_6D_6.

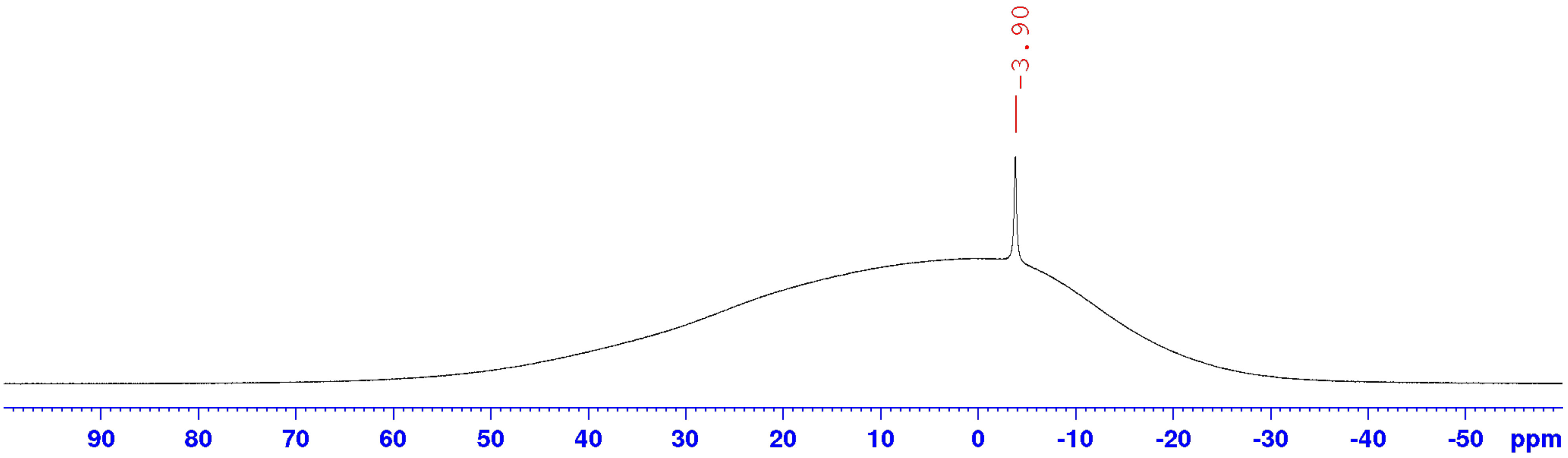

Figure S18. ^{11}B NMR spectrum of **5a** in C_6D_6.

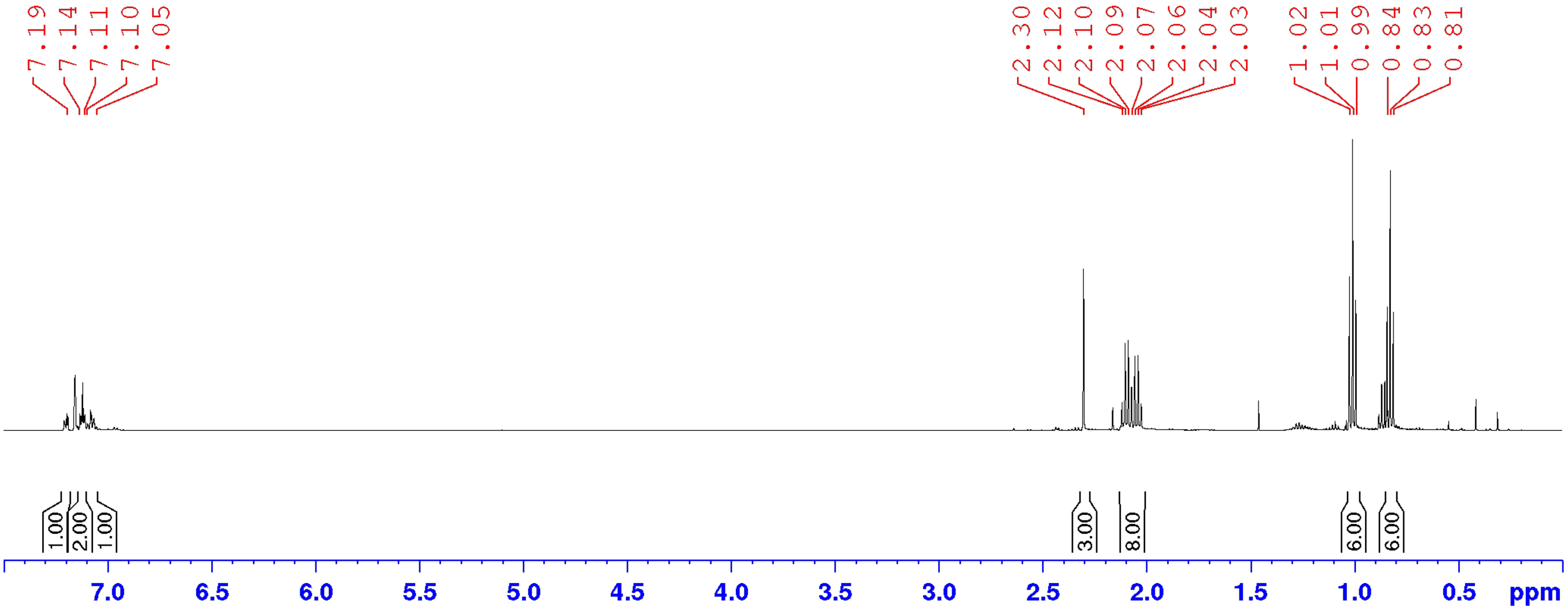

Figure S19. ^{1}H NMR spectrum of **6** in C_6D_6 at 70 °C (Sn route).

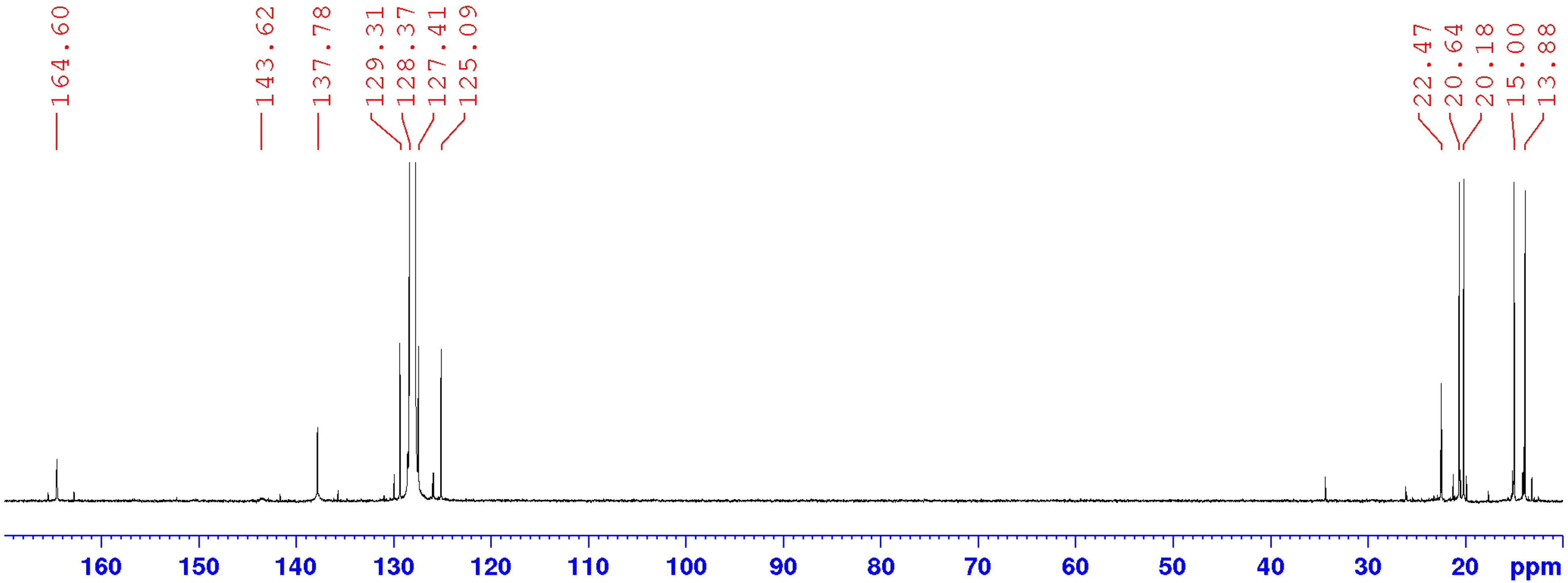

Figure S20. $^{13}C\{^{1}H\}$ NMR spectrum of **6** in C_6D_6 at 70 °C (Sn route).

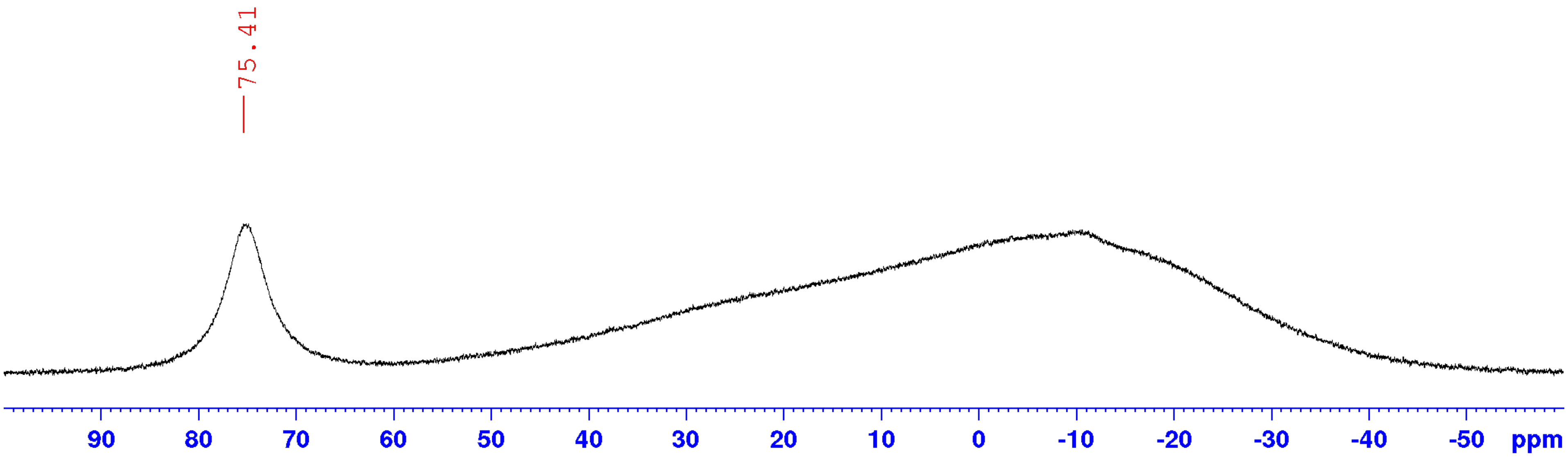

Figure S21. ^{11}B NMR spectrum of **6** in C_6D_6 at 70 °C (Sn route).

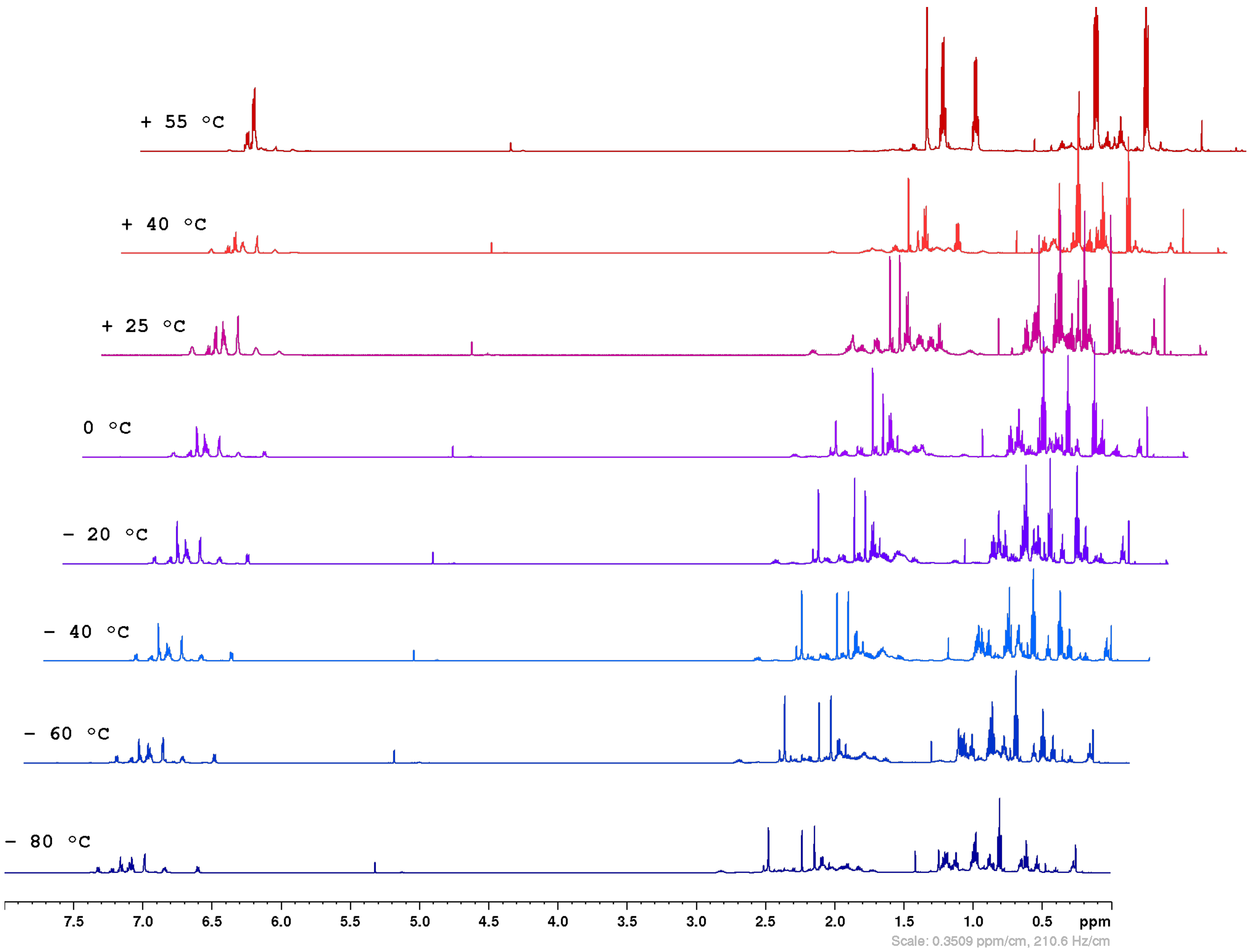

Figure S22. VT ^{1}H NMR of **6** in CD_2Cl_2 (Sn route).

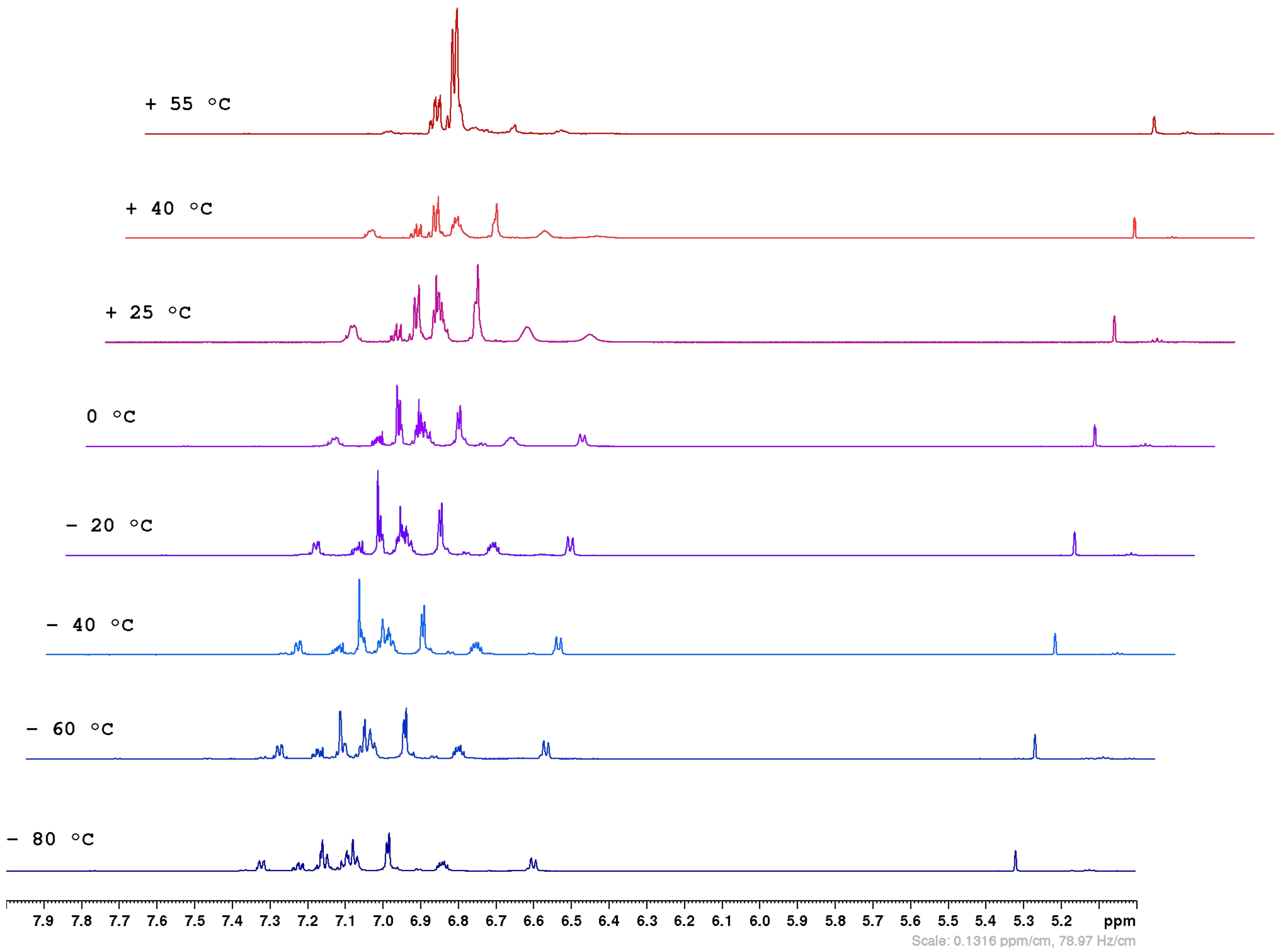

Figure S23. VT ^{1}H NMR of **6** in CD_2Cl_2 with expansion of the aryl region (Sn route).

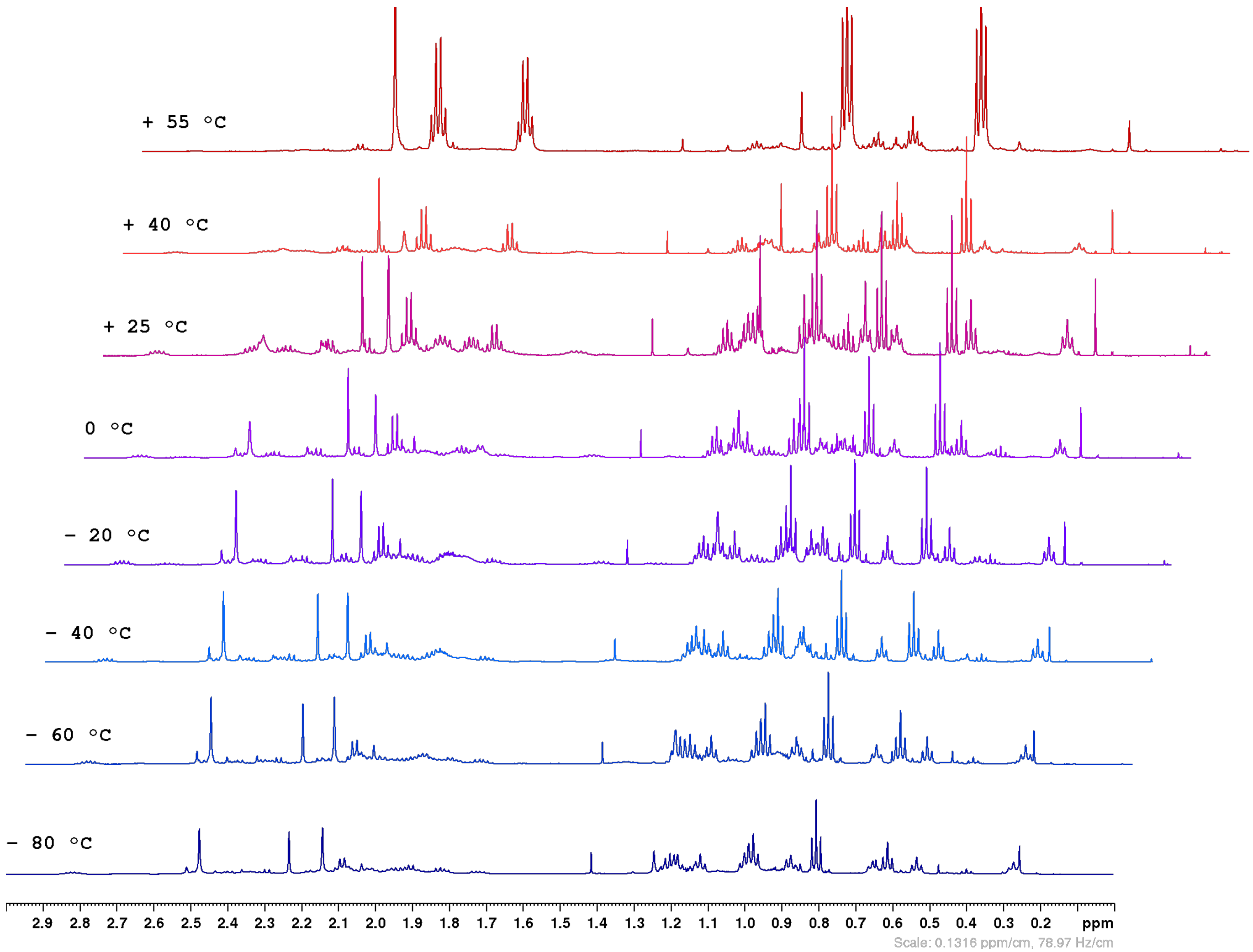

Figure S24. VT ^{1}H NMR of **6** in CD_2Cl_2 with expansion of the aliphatic region (Sn route)

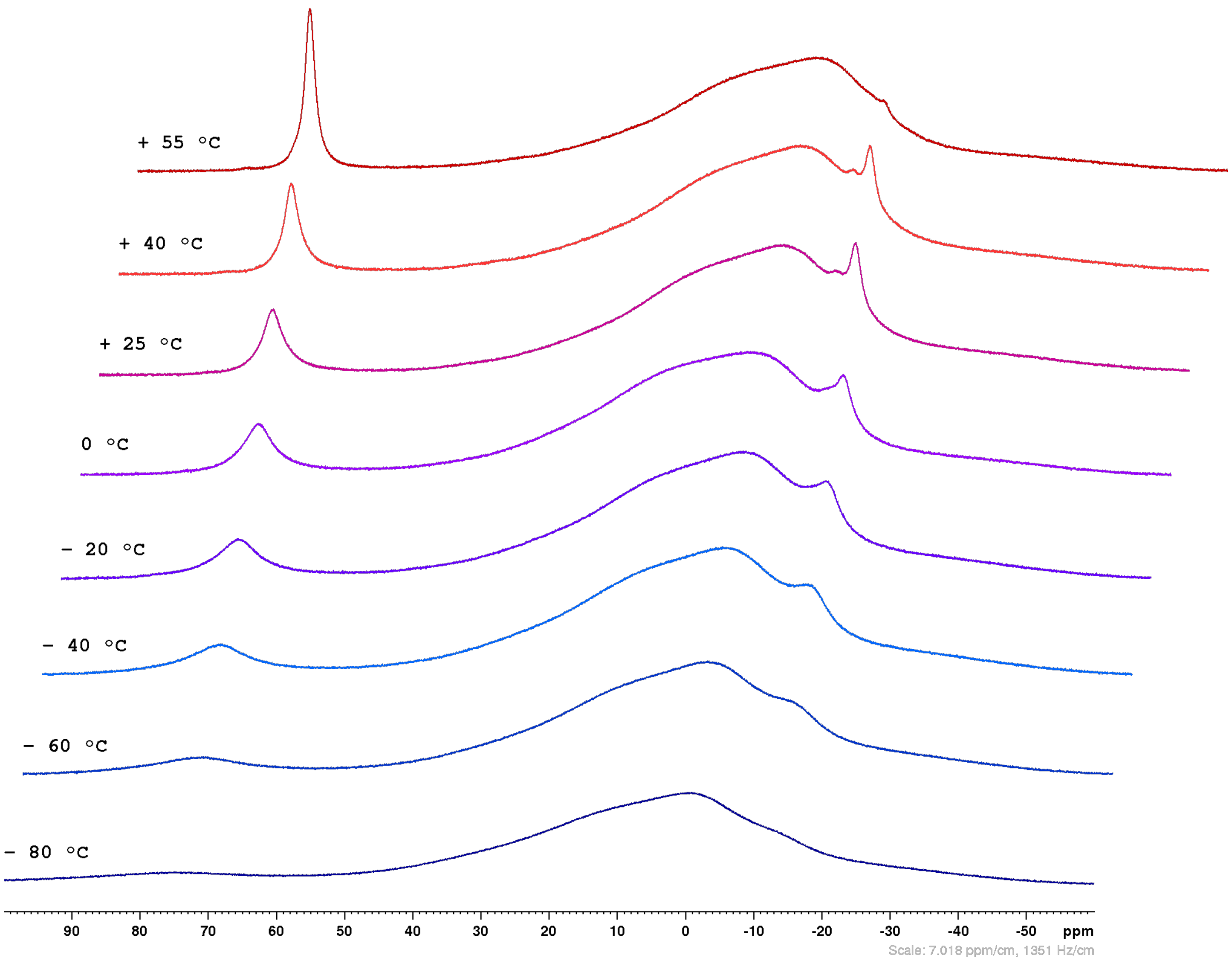

Figure S25. VT ^{11}B NMR of **6** in CD_2Cl_2 (Sn route).

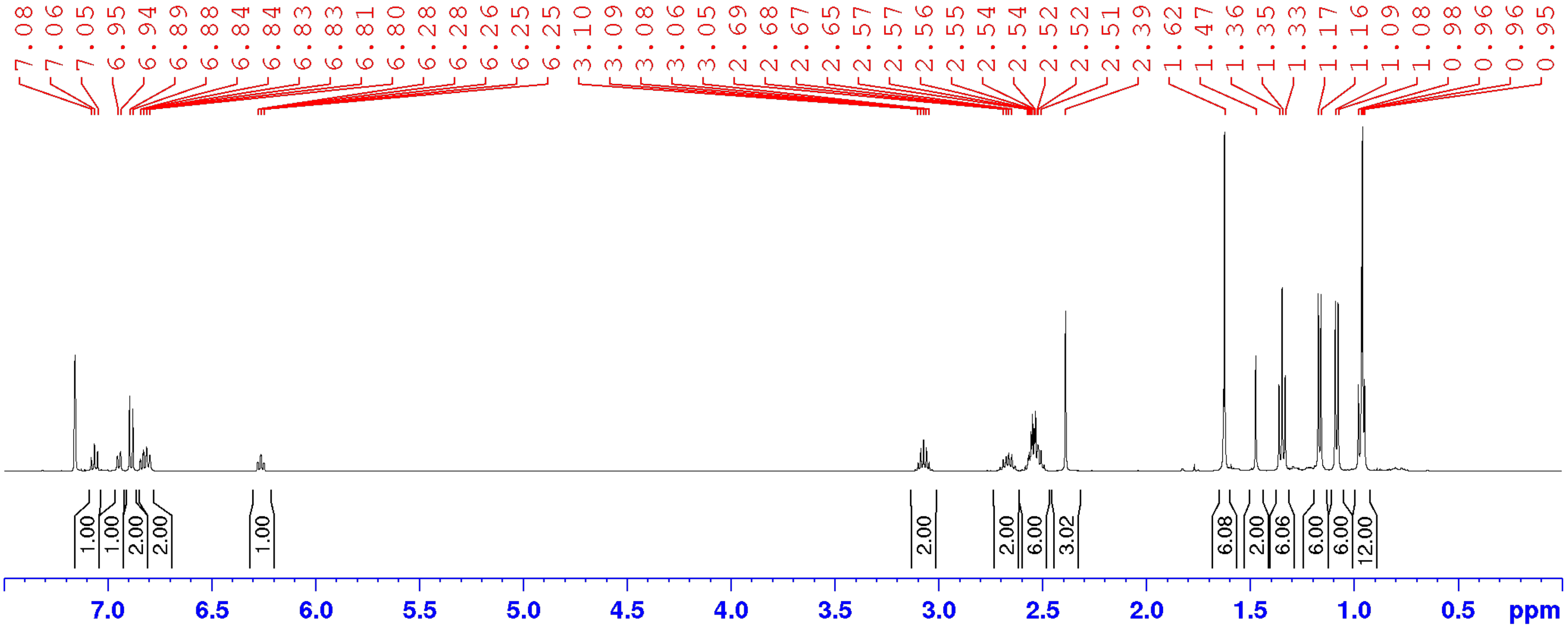

Figure S26. ^{1}H NMR spectrum of **6a** in C_6D_6.

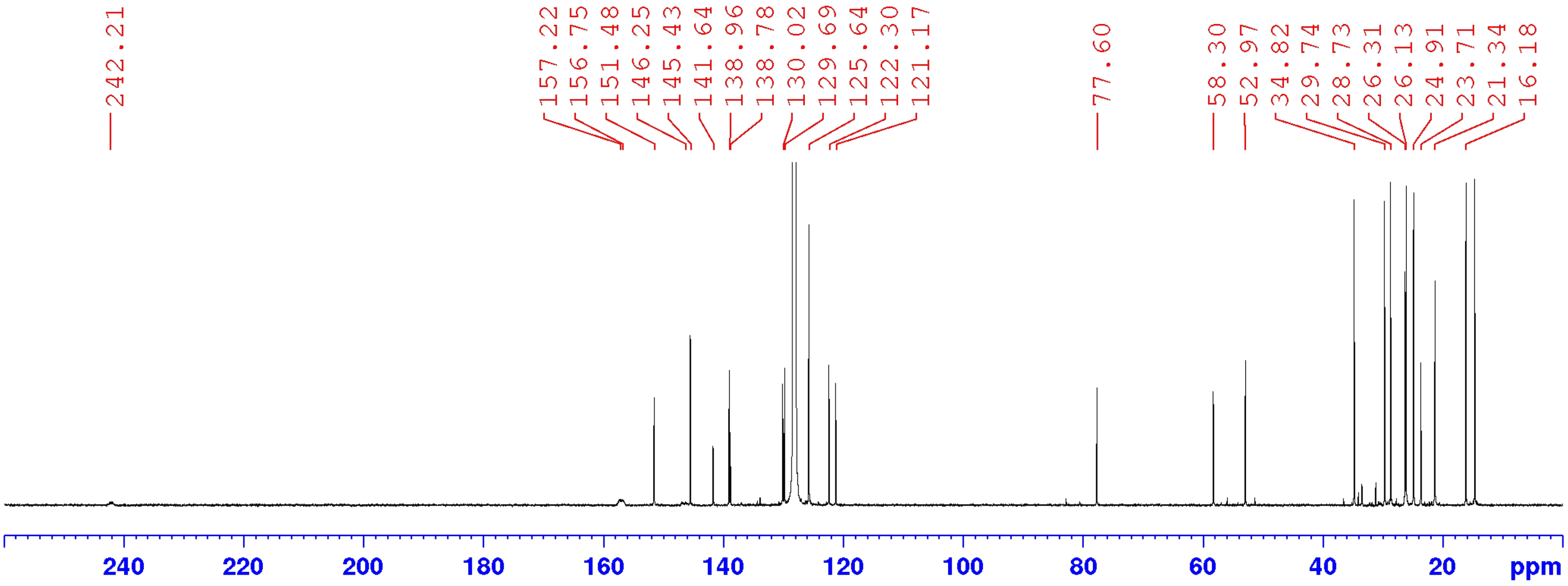

Figure S27. $^{13}C\{^1H\}$ NMR spectrum of **6a** in C_6D_6.

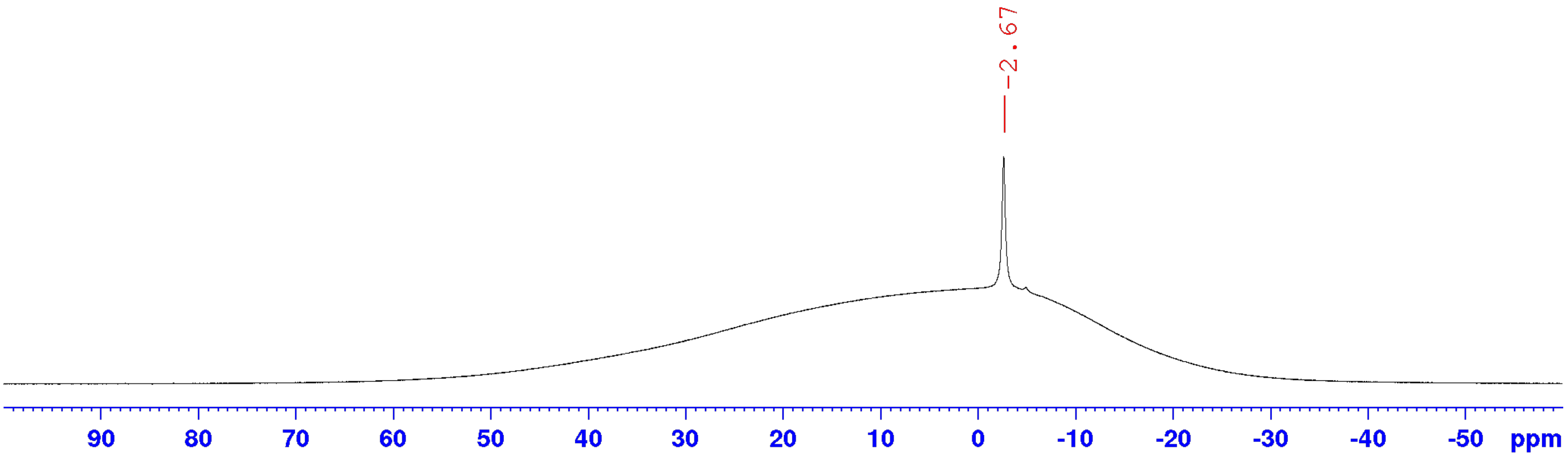

Figure S28. ^{11}B NMR spectrum of **6a** in C_6D_6.

UV-vis spectra

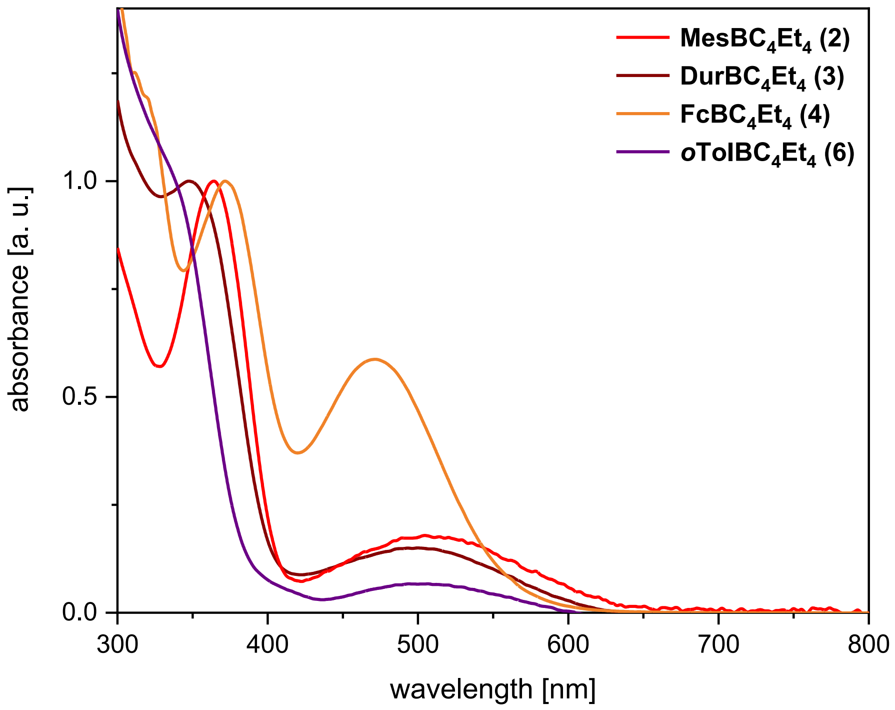

Figure S29. UV-vis absorption spectra of **2** (red), **3** (brown), **4** (orange), and **6** (purple) in DCM at 23 °C.

Table S1. Observed absorption wavelengths [nm] of **2**, **3**, **4** and **6**.

compound	solvent	$\lambda_{max(1)}$	shoulder	$\lambda_{max(2)}$
**2**	DCM	505	--	364
**3**		500	--	347
**4**		472	--	372
**6**		507	337	--

Cyclic voltammetry

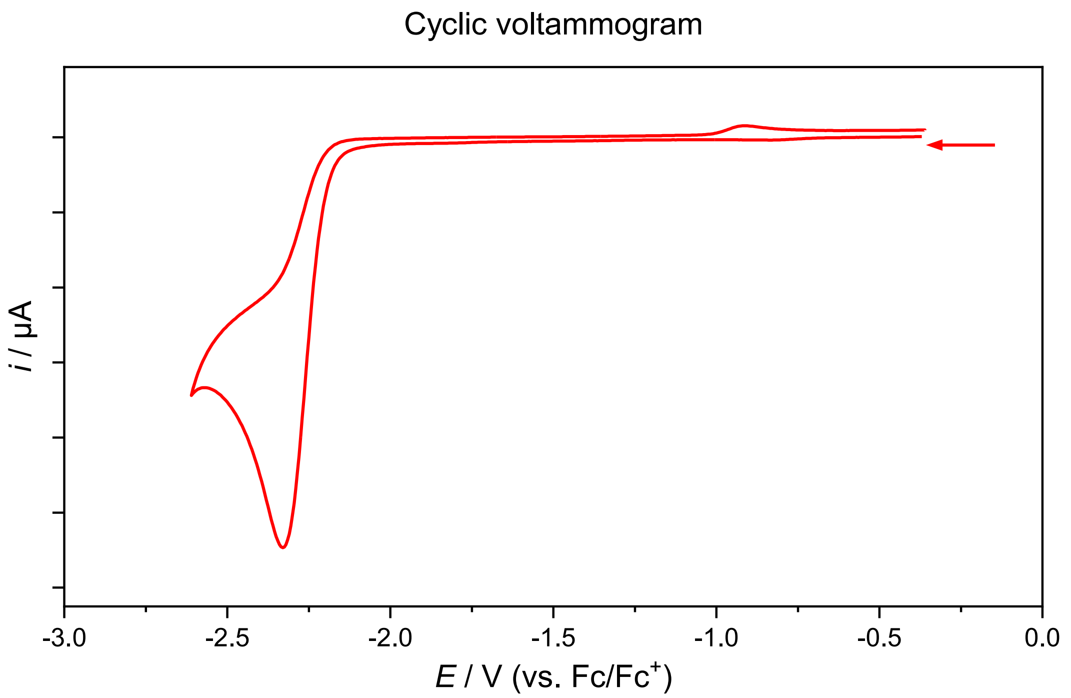

Figure S30. Cyclic voltammogram of **2** in DCM/0.1 M [nBu$_4$N][PF$_6$] measured at 250 mV s^{-1}. Formal reduction potential: E_{pc} = −2.33 V.

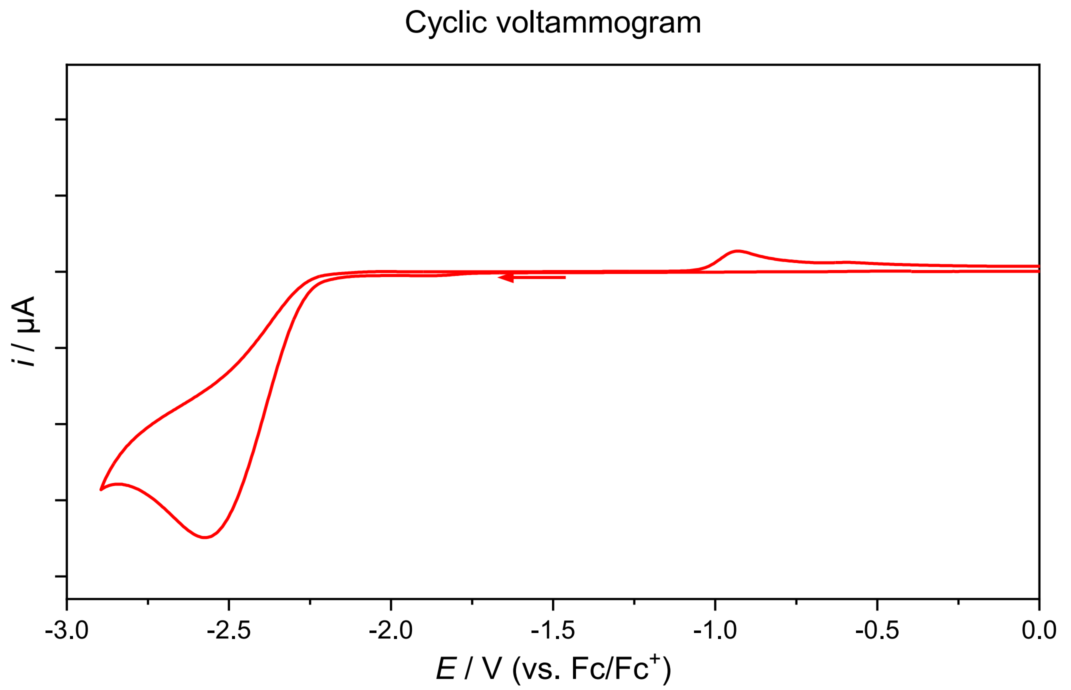

Figure S31. Cyclic voltammogram of **3** in DCM/0.1 M [nBu$_4$N][PF$_6$] measured at 250 mV s^{-1}. Formal reduction potential: E_{pc} = −2.57 V.

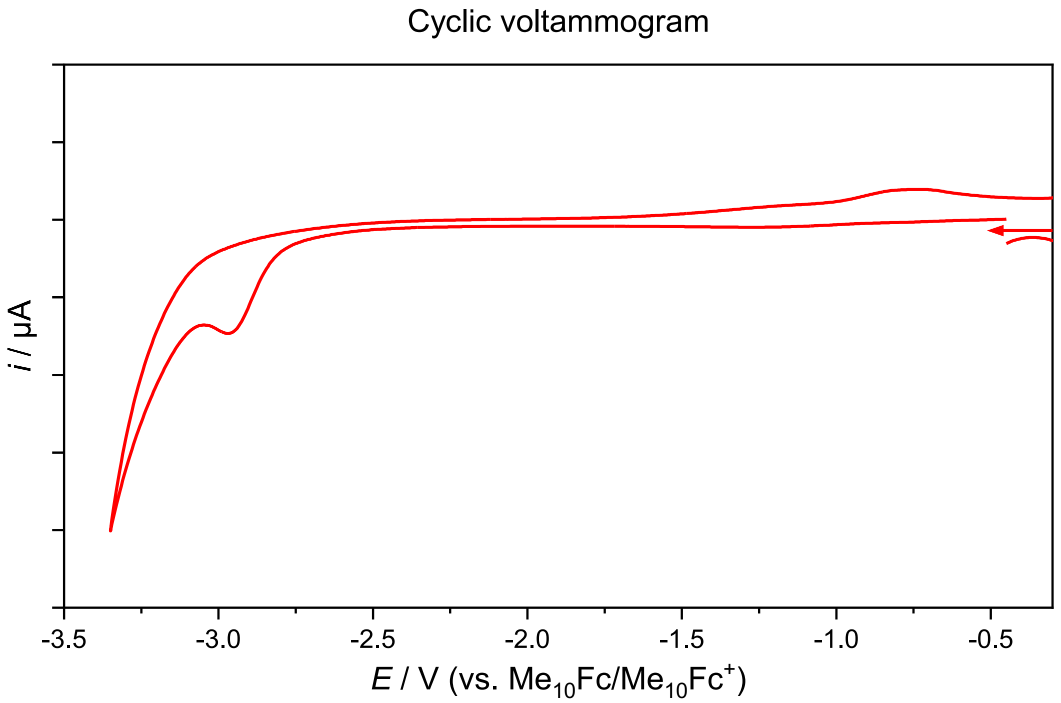

Figure S32. Cyclic voltammogram of **4** in THF/0.1 M [nBu$_4$N][PF$_6$] measured at 250 mV s^{-1}. Formal reduction potential: $E_{pc} = -2.97$ V.

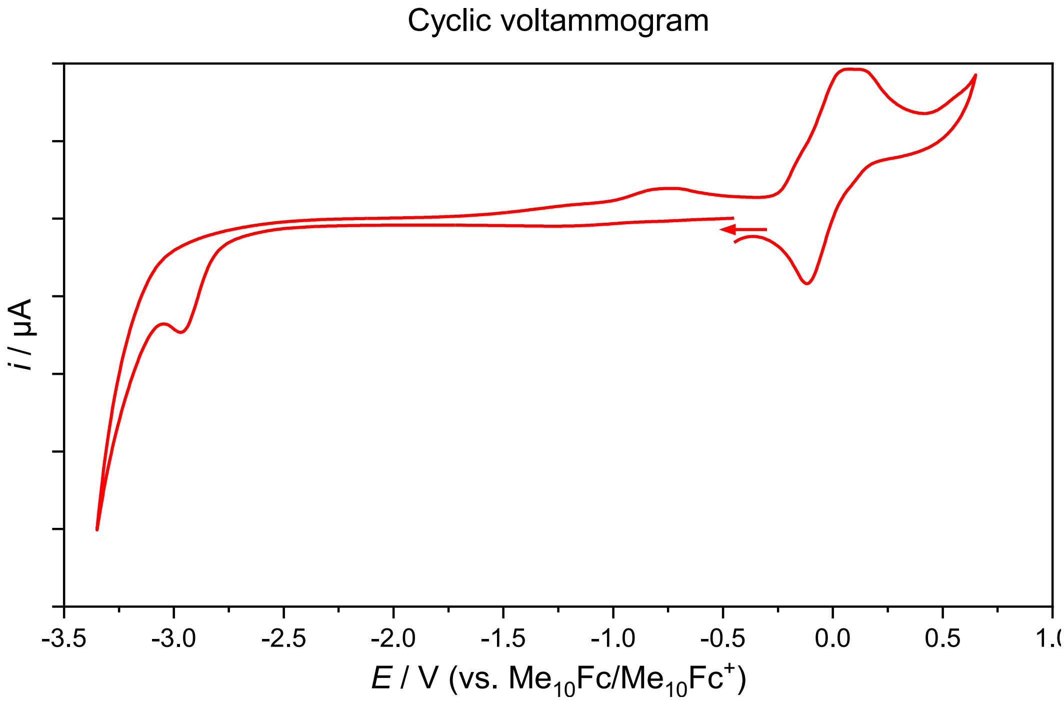

Figure S33. Cyclic voltammogram of **4** in THF/0.1 M [nBu$_4$N][PF$_6$] measured at 250 mV s^{-1} including the oxidation of the ferrocene unit ($E_{1/2}$ = ca. − 0.02 V).

X-ray crystallographic data

The crystal data of **3**, **5a** and **6a** were collected on a RIGAKU XTALAB SYNERGY-R diffractometer with a HPA area detector and multi-layer mirror monochromated $Cu_{K\alpha}$ radiation. The structure was solved using the intrinsic phasing method,[10] refined with the SHELXL program[11] and expanded using Fourier techniques. All non-hydrogen atoms were refined anisotropically.

Refinement details for 3: The molecule was refined as disordered over the mirror plane with local symmetry switched off. The atomic displacement parameters of the atoms were restrained with the RIGU keyword in the ShelXL input ('enhanced rigid bond' restraint for all bonds in the connectivity list). The U_{ii} displacement parameters of all atoms positioned close to the symmetry plane were restrained with the ISOR keyword to approximate isotropic behavior. The atomic displacement parameters of atoms whose symmetry equivalents overlapped were constrained to the same volumes. The distances in the ethyl groups were restrained during refinement to the same value. To keep the axial position of the Br atom in the minor component of the disorder (duryl bromide is a 3% impurity) the 1--3 distances B-C(Dur) were restrained. ADPs of this atom were based on C4 of the duryl group and were kept constrained during refinement. Data were refined as a two-component twin. Twin-law was computed with CrysalisPro during indexing. Less than 1.5% of reflections were overlapping between both twin domains. The BASF parameter was refined to only 0.3%. However, using twin refinement improved the K statistic for weak reflections (K = mean[Fo^2]/mean[Fc^2]).

Crystal data for 3: $C_{22}H_{32.97}BBr_{0.03}$, M_r = 310.66, clear light red block, 0.070×0.040×0.020 mm^3, monoclinic space group $P2_1/m$, a = 9.7239(7) Å, b = 9.0900(5) Å, c = 11.1927(8) Å, α = 90°, β = 97.359(7)°, γ = 90°, V = 981.18(12) Å^3, Z = 2, ρ_{calcd} = 1.052 g·cm^{-3}, μ = 0.488 mm^{-1}, $F(000)$ = 342, T = 100(2) K, R_1 = 0.1138, wR_2 = 0.2398, 2030 independent reflections [$2\theta \leq 147.174°$] and 197 parameters.

Crystal data for 5a: $C_{38}H_{56}BN$, M_r = 537.64, clear light orange block, 0.220×0.120×0.090 mm^3, monoclinic space group $P2_1/c$, a = 16.9907(2) Å, b = 10.55440(10) Å, c = 18.3576(2) Å, α = 90°, β = 96.4760(10)°, γ = 90°, V = 3271.00(6) Å^3,

$Z = 4$, $\rho_{calcd} = 1.092$ g·cm^{-3}, $\mu = 0.448$ mm^{-1}, $F(000) = 1184$, $T = 100(2)$ K, $R_1 = 0.0507$, $wR_2 = 0.1110$, 6637 independent reflections [$2\theta \leq 150.716°$] and 373 parameters.

Crystal data for 6a: $C_{39}H_{58}BN$, $M_r = 551.67$, clear orange block, 0.170×0.080×0.060 mm^3, monoclinic space group $P2_1/c$, $a = 17.1660(2)$ Å, $b = 10.52710(10)$ Å, $c = 18.3364(2)$ Å, $\alpha = 90°$, $\beta = 96.4030(10)°$, $\gamma = 90°$, $V = 3292.87(6)$ Å^3, $Z = 4$, $\rho_{calcd} = 1.113$ g·cm^{-3}, $\mu = 0.457$ mm^{-1}, $F(000) = 1216$, $T = 100(2)$ K, $R_1 = 0.0440$, $wR_2 = 0.1016$, 6242 independent reflections [$2\theta \leq 140.14°$] and 383 parameters.

Crystallographic data have been deposited with the Cambridge Crystallographic Data Center as supplementary publication no. CCDC-2263083 (**3**), CCDC-2263084 (**5a**), and CCDC-2263085 (**6a**). These data can be obtained free of charge from The Cambridge Crystallographic Data Centre *via* www.ccdc.cam.ac.uk/data_request/cif.

Computational details

All calculations were done using the Gaussian 16, Revision C.01[12] quantum chemistry program package. Geometry optimizations for the closed-shell singlet and the triplet states of $MesBC_4Et_4$ (**2**) and $MesBC_4Ph_4$ were performed at the (U)PBE0-D3(BJ)[13-16]/6-31+G(d,p)[17-24] level of theory. All optimized geometries were characterized as minima on the corresponding potential energy surface by vibrational frequency calculations, which revealed that all eigenvalues of the Hessian matrices are positive. The vertical and adiabatic singlet-triplet energy gaps were obtained at the (U)PBE0-D3(BJ)/6-311+G(d,p)[25-26] level of theory (see Table S2).

To assess the aromaticity character of the systems, calculations of the nucleus-independent chemical shift (NICS)[27-29] at the PBE0-D3(BJ)/6-311+G(d,p) level of theory were performed. These were accomplished by using the gauge-independent atomic orbital (GIAO)[30-32] method. The corresponding values were obtained by placing dummy atoms in the centers of BC_4 rings and at distances of 0.1 Å along the axis perpendicular to the ring centers (see Table S3). For the $NICS_{zz}$ scan, the zz component of the magnetic shielding tensor was used (see Figure S34).[33] Furthermore, the anisotropy of the induced current density (ACID)[34-35] method at the PBE0-D3(BJ)/6-311+G(d,p) level of theory was used to further confirm and support the results (see Figure S35). For this only the relevant π-orbitals were considered.

To analyze the bonding situations in the different systems, the Wiberg bond indices (WBIs)[36] and the Mayer bond order (MBOs)[37] as well as natural bond orbital (NBO)[38] charges were obtained at the PBE0-D3(BJ)/6-311G(d,p) level of theory (see Table S4). For the former two the Multiwfn 3.8[39] tool was used and for the last the NBO 7[38] program package.

The frontier orbitals are plotted in Figure S36 and their energetic level is given at the PBE0-D3(BJ)/6-311+G(d,p)/SMD(benzene) level of theory. For graphic representation GaussView 6.0.16[40] was used.

Table S2. Vertical and adiabatic singlet-triplet energy gaps of $MesBC_4Et_4$ and $MesBC_4Ph_4$ at the (U)PBE0-D3(BJ)/6-311+G(d,p) level of theory.

	$MesBC_4Et_4$	$MesBC_4Ph_4$
Vertical singlet-triplet gap	32.5 kcal mol^{-1}	25.8 kcal mol^{-1}
Adiabatic singlet-triplet gap	16.3 kcal mol^{-1}	13.0 kcal mol^{-1}

Table S3. NICS and $NICS_{zz}$ values of $MesBC_4Et_4$ and $MesBC_4Ph_4$ at the (U)PBE0-D3(BJ)/6-311+G(d,p) level of theory.

	$MesBC_4Et_4$	$MesBC_4Ph_4$
NICS(0)	16.38 ppm	14.75 ppm
NICS(–1/+1)	8.66 / 9.01 ppm	8.00 / 8.00 ppm
$NICS_{zz}$(0)	59.10 ppm	52.50 ppm
$NICS_{zz}$(–1/+1)	32.76 / 33.20 ppm	27.74 / 27.76 ppm

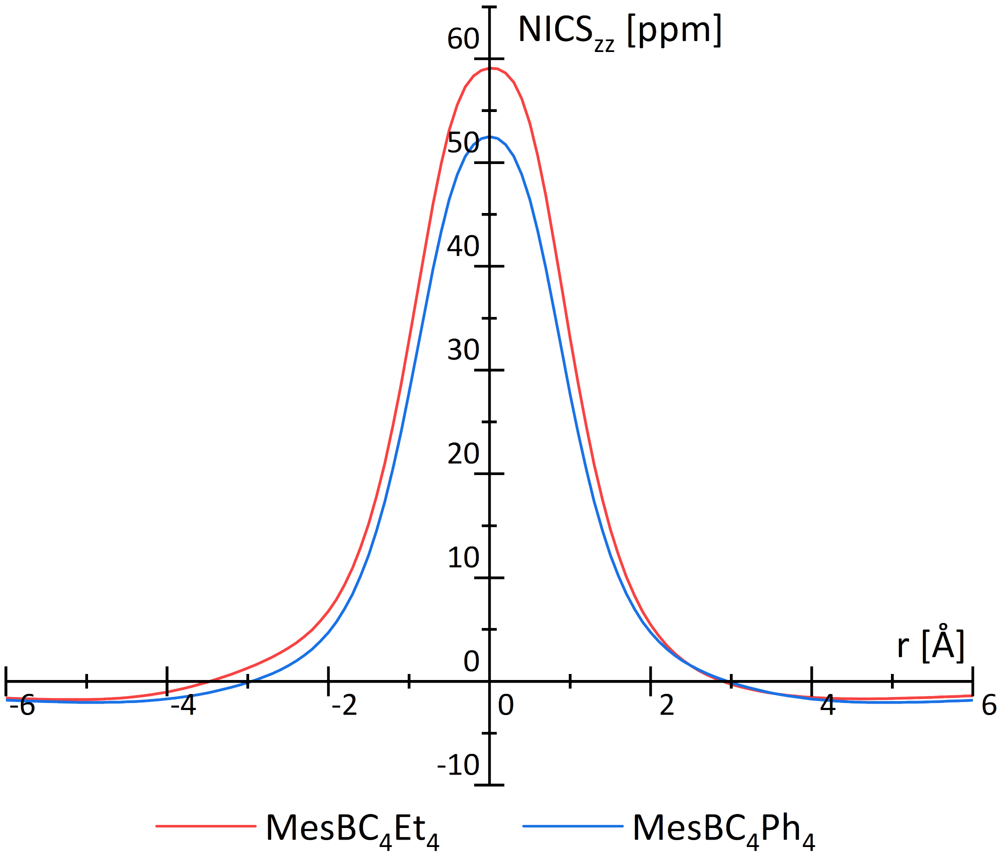

Figure S34. $NICS_{zz}$ scans of $MesBC_4Et_4$ and $MesBC_4Ph_4$.

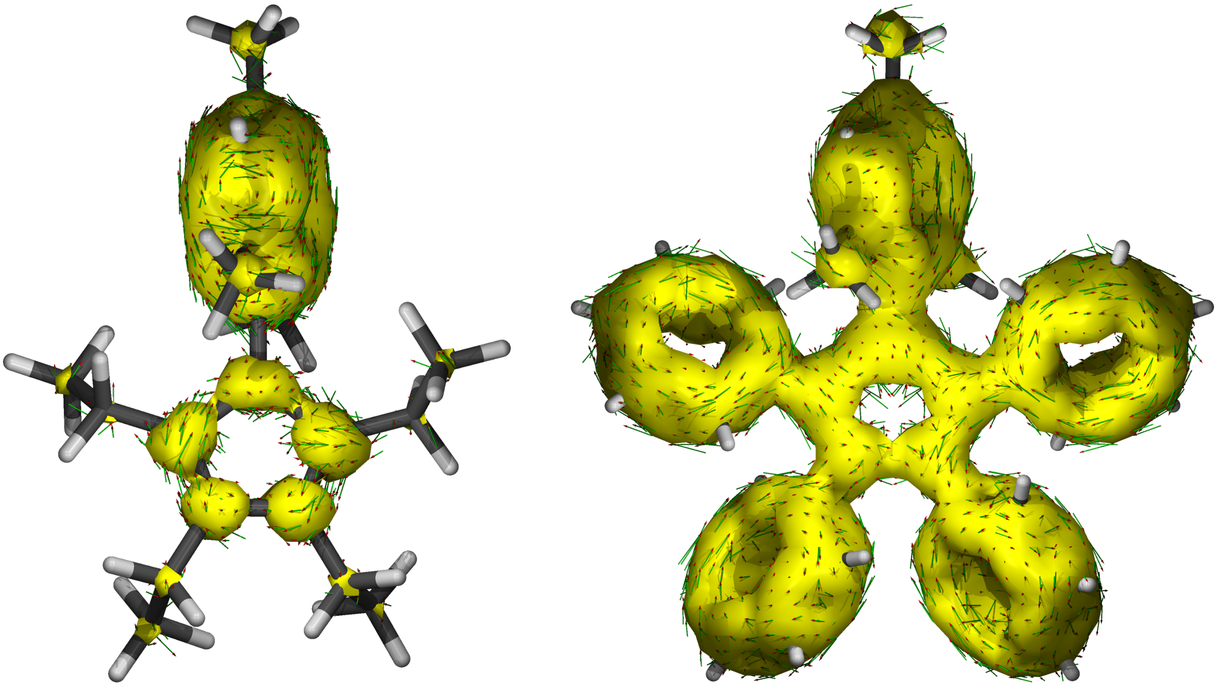

MesBC$_4$Et$_4$ MesBC$_4$Ph$_4$

Figure S35. ACID plots of MesBC$_4$Et$_4$ and MesBC$_4$Ph$_4$. Only the relevant π-orbitals were considered.

Table S4. WBIs, MBOs and NBOs of MesBC$_4$Et$_4$ and MesBC$_4$Ph$_4$ at the PBE0-D3(BJ)/6-311G(d,p) level of theory.

	MesBC$_4$Et$_4$		MesBC$_4$Ph$_4$	
	WBI	MBO	WBI	MBO
B1–C1	0.980	0.900	0.969	0.877
B1–C4	0.985	0.901	0.969	0.877
B1–C5	1.001	0.899	1.020	0.940
C1–C2	1.717	1.875	1.638	1.799
C2–C3	0.966	0.972	0.965	0.959
C3–C4	1.717	1.866	1.638	1.799
	NBO charges		NBO charges	
B1	1.007		1.026	
C1	–0.406		–0.400	
C2	0.057		0.054	

C3	0.057	0.054
C4	–0.410	–0.400

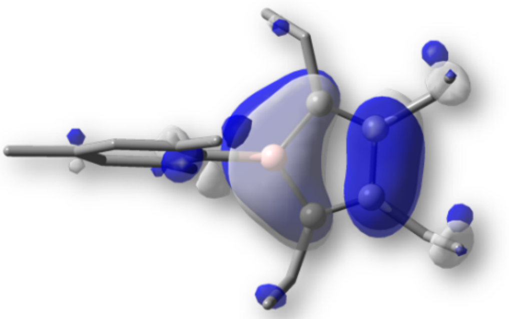

LUMO (E = –2.087 eV)

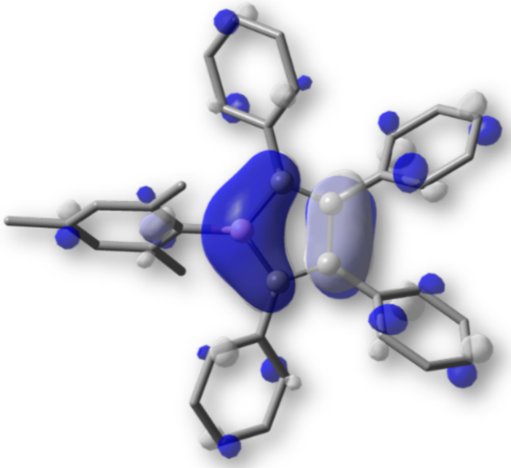

LUMO (E = –2.904 eV)

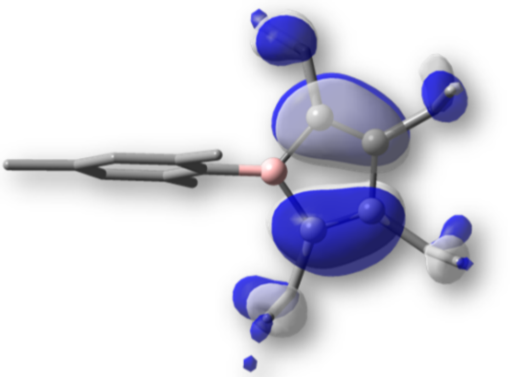

HOMO (E = –5.572 eV)

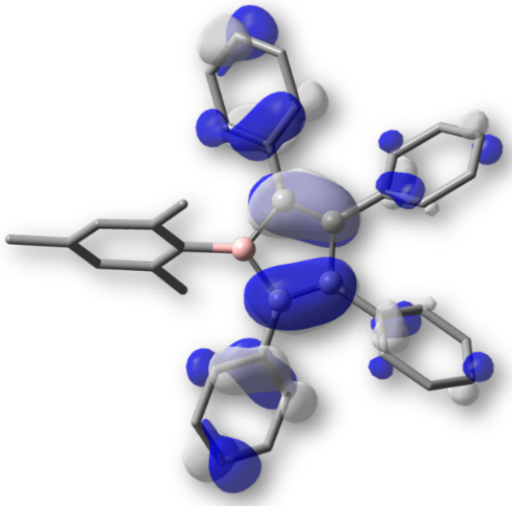

HOMO (E = –5.701 eV)

Figure S36. Frontier orbitals of $MesBC_4Et_4$ (**2**) and $MesBC_4Ph_4$ with their corresponding energetic level at the PBE0-D3(BJ)/6-311+G(d,p)/SMD(benzene) level of theory.

Cartesian Coordinates

Compound 2, PBE0-D3(BJ)/6-31+G(d,p)

Geometry of the lowest singlet state

Energy + Zero-point correction = –842.373701 E_h

C	-1.051364000	1.242111000	-0.501462000
C	-2.319687000	0.771843000	-0.501175000
C	-1.105778000	-1.251515000	-0.155232000
C	-2.352097000	-0.744808000	-0.293777000
B	-0.130459000	-0.013287000	-0.232467000
C	1.415180000	-0.005554000	-0.038124000
C	1.977375000	-0.193341000	1.239259000
C	2.270200000	0.191402000	-1.139244000
C	3.363384000	-0.178528000	1.395338000
C	3.652603000	0.197854000	-0.951820000
C	4.219708000	0.018548000	0.310690000
H	3.786538000	-0.321829000	2.388625000
H	4.303703000	0.339829000	-1.813244000
C	-3.572643000	1.571409000	-0.670733000
C	-4.315381000	1.825189000	0.645332000
H	-4.637044000	0.891866000	1.115689000
C	-3.632807000	-1.526353000	-0.233559000
C	-3.995721000	-1.952961000	1.193869000
H	-3.224185000	-2.605986000	1.611675000
C	-0.730300000	-2.694186000	-0.014257000

C	0.159110000	-3.175514000	-1.163703000
H	1.085039000	-2.593313000	-1.206759000
C	-0.606783000	2.667534000	-0.602104000
C	-0.149119000	3.223087000	0.750299000
H	-0.966798000	3.197535000	1.478571000
C	1.697952000	0.313288000	-2.526264000
H	2.361662000	0.876927000	-3.188626000
H	1.547781000	-0.676924000	-2.974546000
H	0.720860000	0.809521000	-2.518991000
C	1.086067000	-0.343108000	2.443106000
H	1.586396000	-0.887670000	3.249311000
H	0.795852000	0.638222000	2.839244000
H	0.158719000	-0.871874000	2.196303000
C	5.709759000	0.064645000	0.502457000
H	6.045742000	1.085632000	0.720903000
H	6.024202000	-0.568399000	1.337644000
H	6.238633000	-0.268608000	-0.395381000
H	0.236255000	2.740862000	-1.302799000
H	-1.401617000	3.306341000	-1.007030000
H	0.195258000	4.258778000	0.657298000
H	0.678488000	2.628440000	1.150707000
H	-3.323934000	2.533484000	-1.129942000
H	-4.246012000	1.061644000	-1.372937000
H	-5.206294000	2.438322000	0.475605000
H	-3.668659000	2.349911000	1.355364000

H	-4.458494000	-0.947696000	-0.660969000
H	-3.535623000	-2.424467000	-0.856488000
H	-4.948207000	-2.492898000	1.210795000
H	-4.083477000	-1.087851000	1.858092000
H	-1.621905000	-3.330361000	0.047168000
H	0.429730000	-4.229561000	-1.039867000
H	-0.354875000	-3.066077000	-2.124757000
H	-0.183383000	-2.843616000	0.928302000

Compound $MesBC_4Ph_4$, PBE0-D3(BJ)/6-31+G(d,p)

Geometry of the lowest singlet state

Energy + Zero-point correction = –1451.348924 E_h

C	0.046435000	1.255670000	-0.058388000
C	1.313676000	0.761662000	-0.042684000
C	0.048784000	-1.255636000	0.054757000
C	1.315093000	-0.759182000	0.040238000
B	-0.911446000	-0.000887000	-0.002053000
C	-2.463609000	-0.002100000	-0.000787000
C	-3.177772000	-0.533213000	-1.093899000
C	-3.174725000	0.522752000	1.097236000
C	-4.571804000	-0.518509000	-1.078532000
C	-4.568772000	0.497457000	1.090804000
C	-5.287158000	-0.010500000	0.007169000
H	-5.114116000	-0.919982000	-1.933012000
H	-5.108416000	0.887089000	1.952383000

C	2.560882000	1.539366000	-0.102762000
C	3.586526000	1.343128000	0.832633000
C	2.738112000	2.502815000	-1.105914000
C	4.749730000	2.104835000	0.776585000
H	3.466676000	0.595622000	1.610995000
C	3.907887000	3.250729000	-1.170747000
H	1.946350000	2.658863000	-1.832611000
C	4.916479000	3.057274000	-0.226803000
H	5.531175000	1.947017000	1.514329000
H	4.031834000	3.989130000	-1.957718000
H	5.828976000	3.644515000	-0.275269000
C	2.563774000	-1.534399000	0.101949000
C	2.741846000	-2.497191000	1.105581000
C	3.590052000	-1.336357000	-0.832366000
C	3.913046000	-3.242740000	1.171902000
H	1.949595000	-2.654656000	1.831438000
C	4.754736000	-2.095688000	-0.774812000
H	3.469549000	-0.589315000	-1.611075000
C	4.922293000	-3.047510000	0.229026000
H	4.037597000	-3.980684000	1.959206000
H	5.536679000	-1.936498000	-1.511734000
H	5.835913000	-3.632904000	0.278663000
C	-0.361734000	-2.663443000	0.044618000
C	0.307204000	-3.633387000	-0.721448000
C	-1.495287000	-3.062406000	0.771058000

C	-0.131025000	-4.951991000	-0.743943000
H	1.170803000	-3.342404000	-1.311391000
C	-1.929235000	-4.384806000	0.753397000
H	-2.041313000	-2.323292000	1.349674000
C	-1.249054000	-5.336604000	-0.002851000
H	0.399277000	-5.683081000	-1.348036000
H	-2.806718000	-4.669143000	1.327456000
H	-1.590673000	-6.367532000	-0.022620000
C	-0.366912000	2.662624000	-0.047722000
C	-1.500759000	3.059672000	-0.774742000
C	0.299492000	3.633558000	0.719313000
C	-1.937245000	4.381235000	-0.756963000
H	-2.044707000	2.319633000	-1.354131000
C	-0.141320000	4.951299000	0.741995000
H	1.163247000	3.344043000	1.309752000
C	-1.259497000	5.334059000	0.000174000
H	-2.814712000	4.664217000	-1.331734000
H	0.387092000	5.683176000	1.346790000
H	-1.603069000	6.364336000	0.020016000
C	-2.436859000	1.080400000	2.284930000
H	-3.124230000	1.323317000	3.099849000
H	-1.698471000	0.369033000	2.674019000
H	-1.896506000	1.995432000	2.017208000
C	-2.443309000	-1.093794000	-2.282257000
H	-3.133160000	-1.340761000	-3.093857000

H	-1.708022000	-0.381996000	-2.676329000
H	-1.899916000	-2.006742000	-2.013609000
C	-6.789401000	0.014956000	-0.002926000
H	-7.161646000	0.948268000	-0.442718000
H	-7.198711000	-0.810604000	-0.592716000
H	-7.196556000	-0.053892000	1.010122000

References

1 I. Noviandri, K. N. Brown, D. S. Fleming, P. T. Gulyas, P. A. Lay, A. F. Masters and L. Phillips, *J. Phys. Chem. B*, 1999, **103**, 6713-6722.

2 R. Drescher, S. Lin, A. Hofmann, C. Lenczyk, S. Kachel, I. Krummenacher, Z. Lin and H. Braunschweig, *Chem. Sci.*, 2020, **11**, 5559-5564.

3 H. Braunschweig, Q. Ye and K. Radacki, *Chem. Commun.*, 2012, **48**, 2701-2703.

4 W. Haubold, J. Herdtle, W. Gollinger and W. Einholz, *J. Organomet. Chem.*, 1986, **315**, 1-8.

5 W. Schacht and D. Kaufmann, *Chem. Ber.*, 1987, **120**, 1331-1338.

6 S. Zhang, Y. Lu, Y. Li, K. Wang, J. Chen and Z. Zhang, *Org. Lett.*, 2017, **15**, 3986-3989.

7 T. Renk, W. Ruf and W. Siebert, *J. Organomet. Chem.*, 1976, **120**, 1-25.

8 L. Liu, W.-X. Zhang, Q. Luo, H. Li, and Z. Xi, *Organometallics*, 2010, **29**, 278-281.

9 Y. Ura, Y. Li, Z. Xi and T. Takahashi, *Tetrahedron Lett.*, 1998, **39**, 2787-2790.

10 G. Sheldrick, *Acta Cryst.*, 2015, **A71**, 3-8.

11 G. Sheldrick, *Acta Cryst.*, 2008, **A64**, 112-122.

12 Gaussian 16, Revision C.01, M. J. Frisch, G. W. Trucks, H. B. Schlegel, G. E. Scuseria, M. A. Robb, J. R. Cheeseman, G. Scalmani, V. Barone, G. A. Petersson, H. Nakatsuji, X. Li, M. Caricato, A. V. Marenich, J. Bloino, B. G. Janesko, R. Gomperts, B. Mennucci, H. P. Hratchian, J. V. Ortiz, A. F. Izmaylov, J. L. Sonnenberg, D. Williams-Young, F. Ding, F. Lipparini, F. Egidi, J. Goings, B. Peng, A. Petrone, T. Henderson, D. Ranasinghe, V. G. Zakrzewski, J. Gao, N. Rega, G. Zheng, W. Liang, M. Hada, M. Ehara, K. Toyota, R. Fukuda, J. Hasegawa, M. Ishida, T. Nakajima, Y. Honda, O. Kitao, H. Nakai, T. Vreven, K. Throssell, J. A. Montgomery, Jr., J. E. Peralta, F. Ogliaro, M. J. Bearpark, J. J. Heyd, E. N. Brothers, K. N. Kudin, V. N. Staroverov, T. A. Keith, R. Kobayashi, J. Normand, K. Raghavachari, A. P. Rendell, J. C. Burant, S. S. Iyengar, J. Tomasi, M. Cossi, J. M. Millam, M. Klene, C. Adamo, R. Cammi, J. W. Ochterski, R. L. Martin, K. Morokuma, O. Farkas, J. B. Foresman and D. J. Fox, Gaussian, Inc., Wallingford CT, 2016.

13 S. Grimme, J. Antony, S. Ehrlich and H. Krieg, *J. Chem. Phys.*, 2010, **132**, 154104.

14 S. Grimme, S. Ehrlich and L. Goerigk, *J. Comput. Chem.*, 2011, **32**, 1456-1465.

15 J. P. Perdew, K. Burke and M. Ernzerhof, *Phys. Rev. Lett.*, 1996, **77**, 3865-3868.

16 C. Adamo and V. Barone, *J. Chem. Phys.*, 1999, **110**, 6158-6170.

17 R. Ditchfield, W. J. Hehre and J. A. Pople, *J. Chem. Phys.*, 1971, **54**, 724-728.

18 W. J. Hehre, W. Ditchfield and J. A. Pople, *J. Chem. Phys.*, 1972, **56**, 2257-2261.

19 P. C. Hariharan and J. A. Pople, *Theor. Chim. Acta*, 1973, **28**, 213-222.

20 J. D. Dill and J. A. Pople, *J. Chem. Phys.*, 1975, **62**, 2921-2923.

21 R. Krishnan, J. S. Binkley, R. Seeger and J. A. Pople, *J. Chem. Phys.*, 1980, **72**, 650-654.

22 M. M. Francl, W. J. Pietro, W. J. Hehre, J. S. Binkley, M. S. Gordon, D. J. DeFrees and J. A. Pople, *J. Chem. Phys.*, 1982, **77**, 3654-3665.

23 M. S. Gordon, J. S. Binkley, J. A. Pople, W. J. Pietro and W. J. Hehre, *J. Am. Chem. Soc.*, 1982, **104**, 2797-2803.

24 G. W. Spitznagel, T. Clark, P. von Ragué Schleyer and W. J. Hehre, *J. Comput. Chem.*, 1987, **8**, 1109-1116.

25 R. Krishnan, J. S. Binkley, R. Seeger and J. A. Pople, *J. Chem. Phys.*, 1980, **72**, 650-654.

26 T. Clark, J. Chandrasekhar, G. W. Spitznagel and P. von Ragué Schleyer, *J. Comput. Chem.*, 1983, **4**, 294-301.

27 P. von Ragué Schleyer, C. Maerker, A. Dransfeld, H. Jiao and N. J. R. van Eikema Hommes, *J. Am. Chem. Soc.*, 1996, **118**, 6317-6318.

28 Z. Chen, C. S. Wannere, C. Corminboeuf, R. Puchta and P. von Ragué Schleyer, *Chem. Rev.*, 2005, **105**, 3842-3888.

29 J. O. C. Jiménez-Halla, E. Matito, J. Robles and M. Solà, *J. Organomet. Chem.*, 2006, **691**, 4359-4366.

30 R. Ditchfield, *Mol. Phys.*, 1974, **27**, 789-807.

31 K. Wolinski, J. F. Hinton and P. Pulay, *J. Am. Chem. Soc.*, 1990, **112**, 8251-8260.

32 J. R. Cheeseman, G. W. Trucks, T. A. Keith and M. J. Frisch, *J. Chem. Phys.* 1996, **104**, 5497-5509.

33 A. C. Tsipis, *Phys. Chem. Chem. Phys.*, 2009, **11**, 8244-8261.

34 R. Herges and D. Geuenich, *J. Phys. Chem. A*, 2001, **105**, 3214-3220.

35 D. Geuenich, K. Hess, F. Köhler and R. Herges, *Chem. Rev.*, 2005, **105**, 3758-3772.

36 K. B. Wiberg, *Tetrahedron*, 1968, **24**, 1083-1096.

37 I. Mayer, *J. Comput. Chem.*, 2007, **28**, 204-221.

38 F. Weinhold, C. R. Landis and E. D. Glendening, *Int. Rev. Phys. Chem.*, 2016, **35**, 399-440.

39 T. Lu and F. Chen, *J. Comput. Chem.*, 2012, **33**, 580-592.

40 R. Dennington, T. Keith and J. Millam, GaussView, Version 6.0.16, Shawnee Mission, KS, 2016.
